# Soil Moisture‐Cloud‐Precipitation Feedback in the Lower Atmosphere From Functional Decomposition of Satellite Observations

**DOI:** 10.1029/2024GL110347

**Published:** 2024-11-21

**Authors:** Yifu Gao, Clément Guilloteau, Efi Foufoula‐Georgiou, Chonggang Xu, Xiaoming Sun, Jasper A. Vrugt

**Affiliations:** ^1^ Department of Civil and Environmental Engineering University of California Irvine CA USA; ^2^ Earth and Environmental Sciences Division Los Alamos National Laboratory Los Alamos NM USA

**Keywords:** soil moisture, precipitation, land‐atmosphere interaction, hydroclimatology, United States

## Abstract

The feedback of topsoil moisture (SM) content on convective clouds and precipitation is not well understood and represented in the current generation of weather and climate models. Here, we use functional decomposition of satellite‐derived SM and cloud vertical profiles (CVP) to quantify the relationship between SM and the vertical distribution of cloud water in the central US. High‐dimensional model representation is used to disentangle the contributions of SM and other land‐surface and atmospheric variables to the CVP. Results show that the sign and strength of the SM‐cloud‐precipitation feedback varies with cloud height and time lag and displays a large spatial variability. Positive anomalies in antecedent 7‐hr SM and land‐surface temperature enhance cloud reflectivity up to 4 dBZ in the lower atmosphere about 1–3 km above the surface. Our approach presents new insights into the SM‐cloud‐precipitation feedback and aids in the diagnosis of land‐atmosphere interactions simulated by weather and climate models.

## Key Points

- Functional decomposition of satellite‐measured soil moisture (SM) and cloud vertical profiles (CVP) provides insights into SM‐CVP feedbacks
- The sign and strength of this feedback varies with height, time lag, and geographic location, in agreement with qualitative studies
- Our approach can be used to diagnose weather and climate model biases as they relate to land‐atmosphere coupling

## 1 Introduction

Feedbacks between soil moisture (SM) and precipitation regulate regional hydroclimatic variability. These feedbacks are determined by a large number of variables and processes, including (variations in) land surface temperature (Koster et al., 2006), energy partitioning (Fast et al., 2019; Golaz et al., 2001; Sakaguchi et al., 2022), and planetary boundary layer (PBL) development (Ek & Holtslag, 2004; Han et al., 2019). In this paper, we focus our attention on diurnal SM‐cloud‐precipitation feedbacks, abbreviated SMCPF, which exert control on the vertical cloud‐water distribution and, consequently, influence weather conditions (Koster et al., 2004) and regional hydroclimatology (Ford et al., 2023; Krakauer et al., 2010; Yin et al., 2014). Much research has been devoted to estimating the sign, causality, and physical linkage of the SMCPF. That research may be divided into simulation‐based analysis (Findell & Eltahir, 2003a; Gentine et al., 2013; Hohenegger et al., 2009; Schär et al., 1999; Schlemmer et al., 2012; Tawfik et al., 2015; G. Wang et al., 2007), observation‐based studies (Ferguson & Wood, 2011; Ford, Rapp, Quiring, & Blake, 2015; Guillod et al., 2015; Santanello et al., 2009; Taylor & Ellis, 2006; Taylor et al., 2010, 2011) and a combination thereof (Baker, Castilho de Souza et al., 2021; Baker, Garcia‐Carreras et al., 2021; Miralles et al., 2014; Santanello et al., 2013; Seneviratne et al., 2006; Spennemann et al., 2018). While numerical models of land‐atmosphere interactions have advanced considerably in recent decades, the sign and strength of simulated SMCPFs are subject to large uncertainties, due to, for instance, the choice of boundary conditions (Hohenegger et al., 2009) and sub‐grid parameterizations (Deardorff, 1980; Thompson et al., 2004, 2008). In observational studies, it is typically difficult to filter out the effects of synoptic variability. In the absence of high‐quality spatiotemporal measurements of SM and cloud vertical profiles, past studies have primarily focused on how SM affects convection initiation, the PBL height, and precipitation probability (Findell et al., 2011; Ford et al., 2023; Frye & Mote, 2010; Graf et al., 2021; Su & Dickinson, 2017; Taylor, 2015; Yuan et al., 2020) without considering diurnal relationships between antecedent SM and the cloud water distribution. Advances in our understanding of SM‐cloud‐precipitation relationships will improve diagnosis of weather and climate model biases and enhance the accuracy of their future projections (Williams, 2019).

Remote‐sensing data products of SM and cloud vertical profiles from polar‐orbiting Earth‐observing satellites have advanced considerably in the past decades and have the potential to substantially advance our understanding of SM‐cloud‐precipitation relationships. Specifically, the 3‐hourly/9 km Soil Moisture Active Passive (SMAP/L4) and 1.5‐hourly/5 km Global Precipitation Measurement Dual‐Frequency Precipitation Radar (GPM/DPR/L2A) provide high‐resolution estimates of the topsoil moisture content and the vertical distribution of hydrometeors within and above the PBL, respectively, at a global coverage. Many studies have confirmed the accuracy and reliability of SMAP/L4 (Koster et al., 2018; Reichle et al., 2017; Tavakol et al., 2019; L. Zhang et al., 2017; X. Zhang et al., 2017) and GPM/DPR/L2A (Lasser et al., 2019; Liao & Meneghini, 2022; Pejcic et al., 2020) data products.

In this paper, we use functional decomposition of a large database of collocated SMAP/L4 surface SM and GPM/DPR/L2A cloud vertical profiles (CVPs) to investigate the functional relationship between antecedent SM and the vertical distribution of cloud water and reflectivity in the lower troposphere. Our method, called high‐dimensional model representation or HDMR (Gao et al., 2023; Li & Rabitz, 2010, 2012) expresses all variable interactions in a hierarchical order and uses the superposition principle to disentangle individual and cooperative contributions of SM and other land‐surface variables to the CVP. We are particularly interested in the so‐called first‐order component functions as they quantify each land‐surface variable's direct contribution to the CVP. As byproduct, HDMR yields maps for our study area of the SM contribution to cloud reflectivity and rainfall, as a function of cloud height and SM time lag. Note that the CVP state decomposition is necessarily incomplete as (a) CVPs are highly variable due to turbulent local dynamics (Heinze et al., 2017) and (b) we consider only a handful of governing variables. The resulting component functions are not intended for CVP prediction, hence we do not report summary statistics of the quality of fit of the HDMR decomposition in the remainder of this paper. The coefficient of determination is low ($R^{2}=0.14$ for HDMR and $R^{2}=0.05$ for linear regression). Rather, we aim to unravel the contribution of each macroscopic land‐surface and atmospheric variable to the CVP, which can be robust and statistically significant when analyzing sufficient observational samples. This can help diagnose weather and climate model biases.

This paper is organized as follows. Section 2 discusses the SMAP/L4 SM and GPM/DPR/L2A satellite products and study region. Section 3 describes the data preprocessing steps and briefly reviews the HDMR method. Section 4 presents the results of our analysis and documents the relationship between SM and the CVP as a function of cloud height, time lag, and location in our study region. Section 5 summarizes our main findings and presents suggestions for future work.

## 2 Data and Experimental Region

We use the publicly available 3‐hourly/9 km SMAP/L4 (March 2015‐present) and 1.5‐hourly/5 km GPM/DPR/L2A (March 2014‐present) data products and select samples from our study region in the warm seasons (April to October) from 2016 to 2019, focusing on convective precipitation in the afternoon hours until midnight (14:00‐24:00 CDT). The altitude extends from 1 to 5 km with Findell and Eltahir (2003a) identifying the 1–3 km zone as a critical region for convective triggering. We briefly discuss the SMAP/L4 and GPM/DPR/L2A products and our study region. A more detailed description of the satellite data products is found in Text S1 in Supporting Information S1.

**Figure 1:**
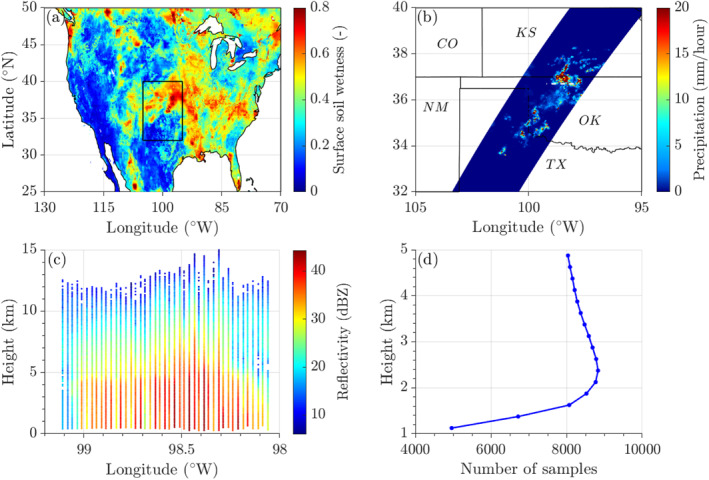
7 August 2016: (a) SMAP/L4 surface SM (3‐hourly, 9 km, 19:30 CDT) over CONUS and GPM/DPR/L2A measured (b) surface precipitation (1.5‐hourly, 5 km, 21:51:10‐23:23:44 CDT) and (c) cloud reflectivity profiles (98.0°W ‐ 99.2°W, 36.7°N) for our study region (black rectangle) in the central United States. The location of the cloud profile is marked using a white dashed line in panel (b). Panel (d) in the bottom right corner displays the sample size n (2016–2019) for each DPR measurement height after data preprocessing has concluded.

The SMAP mission Level 4 SM (L4_SM) product gives 3‐hourly estimates of surface (0–5 cm) SM (see Figure 1a), root‐zone SM, and other land‐surface variables at 9‐km spatial resolution and global coverage (Reichle et al., 2015). As our preliminary data analysis showed that cloud reflectivity was more responsive to topsoil SM than to root‐zone SM, we chose topsoil SM as the key input variable in our CVP functional decomposition. The reasons for this are a stronger diurnal variation and larger root density at the surface (Oerter et al., 2021). As auxiliary land variables, we consider land‐surface temperature (LST) and leaf area index (LAI). In addition, hourly estimates of low‐level atmospheric temperature (AT) and total precipitable water (TPW) from 0.25°×0.25° ERA‐5 reanalysis are adopted as precursors to mesoscale convective events (Findell & Eltahir, 2003a; Holloway & Neelin, 2010; Sherwood, 1999). We use the mean AT for the critical region, 1–3 km above the soil surface. Section 3.2 discusses in more detail our selection of auxiliary land‐surface and atmospheric variables.

The GPM/DPR/L2A product (GPM_2ADPR) provides a swath of precipitation profiles (see Figure 1b) every 1.5 hr at a horizontal resolution of 5 km and vertical increment of 125 m. The major data fields ’zFactorFinal (dBZ)’ and ’typePrecip’ provide vertical profiles of Ka‐band cloud reflectivity (see Figure 1c) and an 8‐digit precipitation type ID. We only select samples classified as convective precipitation and use 250‐m vertically averaged cloud reflectivities to minimize the impact of measurement errors. This averaging demonstrated to be rather inconsequential in our analysis.

Our study region is displayed in Figure 1a (95°W−105°W, 32°N−40°N) and is a hot spot for SM‐precipitation coupling (Findell & Eltahir, 2003b; Ford et al., 2023; Koster et al., 2004) with large spatial variability in climatological sign and strength of the SMCPF (Findell et al., 2011; Ford et al., 2023; Frye & Mote, 2010; Su & Dickinson, 2017; Yuan et al., 2020). This central US region offers an excellent demonstration of our methodology and allows us to benchmark the inferred patterns of the SMCPF sign and magnitude against literature findings.

## 3 Method

### 3.1 Data Preprocessing

We extract the GPM/DPR/L2A swaths that overpass our study region and single out samples classified as convective precipitation in the ’typePrecip’ data field. This type classification is crucial to an accurate characterization of the antecedent atmosphere using ERA‐5 reanalysis AT and TPW data. To avoid water from interception evaporation, we discard samples which, in the 18 hr leading up to the DPR's scan, received more than 0.5 mm of precipitation according to the Multi‐Radar Multi‐Sensor (MRMS) Gauge‐corrected Quantitative Precipitation Estimates (J. Zhang et al., 2016). This should also reduce the impact of large‐scale synoptic systems (Findell et al., 2011). Next, we project SMAP/L4 and ERA‐5 data onto GPM/DPR/L2A coordinates using linear interpolation. Time lags $\Delta t=t_{\text{dpr}}-t_{\text{smap}}$ or $\Delta t=t_{\text{dpr}}-t_{\text{era}\text{5}}$ of 7 and 10 hr are chosen, where $t_{\text{dpr}}$ is the DPR scan time, $t_{\text{smap}}$ denotes the midpoint of the 3‐hr averaging interval, and $t_{\text{era}\text{5}}$ is the ERA‐5 time of hourly snapshots of the atmospheric conditions. In doing so, we allow for a 2‐hr grace period so as to maximize the sample size n at each DPR measurement height. For example, SM data with a time lag 6.01≤Δt≤7.99 are pooled together in the 7‐hr time lag. The two SM time lags find support by our treatment of the synoptic effect (Δt<18 hr), and are in agreement with data‐driven studies on the diurnal SMCPF (Findell et al., 2011; Welty & Zeng, 2018). Figure 1d displays the number of DPR‐measured cloud reflectivities n in the months of April–October (2016–2019) as a function of cloud height. The sample size is not constant with height due to for instance, cloud absence, radar detection limitations and path attenuation (Iguchi et al., 2010). Nevertheless, the pooled reflectivities from April–October guarantee a sufficiently large sample size at each cloud height. Next, we decompose this final collection of SMAP/L4 ‐ GPM/DPR/L2A samples using HDMR and expand the DPR‐measured cloud reflectivities at each cloud height as a sum of first‐ and higher‐order structural and correlative contributions of SM and the auxiliary variables.

### 3.2 High‐Dimensional Model Representation

SMCPFs are notoriously difficult to observe outside of model environments (Ford et al., 2023), hence innovative analytical approaches are required to study them (Berg et al., 2013; Findell et al., 2011; Guillod et al., 2014; Knist et al., 2017; Koster et al., 2004; Seneviratne et al., 2006). HDMR is particularly appealing in this context. Unlike methods such as multivariate linear regression and correlation analysis (Ford et al., 2023; G. Wang et al., 2024; Welty & Zeng, 2018), HDMR accounts explicitly for correlation among the input variables (which is common) and expresses all variable interactions in a hierarchical order. This allows us to disentangle direct and cooperative contributions of individual and groups of dependent input variables to the CVP.

Suppose we group all land‐surface and atmospheric variables that govern cloud reflectivity y=f(x) at a given altitude in a d×1 vector $x={\left(x_{1},\text{…},x_{d}\right)}^{⊤}$. HDMR builds on the multivariable function expansion of Soboľ. (1993) and decomposes the output, y=f(x), of a scalar‐valued square‐integrable function, $f\in L^{2}\left(K^{d}\right)$, on the d‐dimensional unit cube, $K^{d}=\left\{x|0\leq x_{i}\leq 1;\;i=1,\text{…},d\right\}$, into summands of component functions, $f_{i}\left(x_{i}\right)$, $f_{ij}\left(x_{i},x_{j}\right)$, …, $f_{12\text{…}d}\left(x_{1},\;x_{2},\;\text{…},\;x_{d}\right)$, to yield (Li & Rabitz, 2012)

(1) $$y=f_{0}+\sum_{i=1}^{n_{1}}f_{i}\left(x_{i}\right)+\;\;\;\;\sum_{1\leq i<j\leq d}^{n_{2}}\;\;\;\;f_{ij}\left(x_{i},x_{j}\right)+\;\;\;\;\;\;\;\sum_{1\leq i<j<k\leq d}^{n_{3}}\;\;\;\;\;\;\;f_{\mathrm{ijk}}\left(x_{i},x_{j},x_{k}\right)+\text{⋯}+f_{12\text{…}d}\left(x_{1},\;x_{2},\text{…},x_{d}\right)+\epsilon ,$$

where $f_{0}$ denotes the mean output and $\epsilon ∼N\left(0,\sigma_{\epsilon }^{2}\right)$ is a normally distributed residual with zero‐mean and constant variance, $\sigma_{\epsilon }^{2}$. The $n_{1}=d$ first‐order functions, $f_{i}\left(x_{i}\right)$, characterize the individual effects of the input variables on cloud reflectivity, y. The $n_{2}=d\left(d-1\right)/2$ second‐, $f_{ij}\left(x_{i},x_{j}\right)$, $n_{3}=d\left(d-1\right)\left(d-2\right)/6$ third‐, $f_{\mathrm{ijk}}\left(x_{i},x_{j},x_{k}\right)$, up to d ^th^‐order component functions, $f_{12\text{…}d}\left(x_{1},\;x_{2},\text{…},x_{d}\right)$, characterize the cooperative contributions of two, three, up to all land‐surface variables combined to y. In physical systems, third‐ and higher‐order effects are usually negligible (Falchi et al., 2018; Gao et al., 2023; Kucherenko et al., 2011; Rabitz & Aliş, 1999; Shereena & Rao, 2019; H. Wang et al., 2017) and, therefore, we consider only the $n_{12}=n_{1}+n_{2}$ first‐ and second‐order component functions in our CVP function expansion

(2) $$y=f_{0}+\sum_{u=1}^{n_{12}}f_{u}+\epsilon ,$$

where subscript u stands for component function index rather than its order as in Equation 1. Thus, $f_{0}$ signifies the mean reflectivity in units of dBZ, and $f_{1},\text{…},f_{d}$, and, $f_{d+1},\text{…},f_{d+d\left(d-1\right)/2}$, are the first‐ and second‐order component functions, respectively, which quantify the individual and bivariate contributions of the land‐surface and atmospheric variables to cloud reflectivity, y.

We follow Gao et al. (2023) and write the first‐ and second‐order component functions of Equation 2 as a sum of linear multiples of orthonormalized polynomial functions of degrees 1 to p=3 (Li & Rabitz, 2012). This formulation with extended bases and expansion coefficients (linear multiples) helps satisfy a so‐called relaxed vanishing condition (Hooker, 2007)

(3) $$\int_{0}^{1}w_{u}\left(x_{u}\right)f_{u}\left(x_{u}\right)dx_{i}=0\;\text{for}\;\text{all}\;u\subseteq \left\{1,\text{…},d\right\}\;\text{and}\;i\in u,$$

where u is a subset of superset U={1,…,d}, $x_{u}$ denotes the dimensions u of the input vector and $w_{u}\left(x_{u}\right)$ is the probability density function (pdf) of $x_{u}$. The vanishing condition (3) dictates that a second‐order component function, $f_{ij}\left(x_{i},x_{j}\right)$ must be orthogonal to its lower order component functions, $f_{i}\left(x_{i}\right)$ and $f_{j}\left(x_{j}\right)$, and guarantees an exact delineation of the structural and correlative contributions of single and groups of input variables to y (Gao et al., 2023; Li & Rabitz, 2012). D‐MORPH regression (Li & Rabitz, 2010) enforces the vanishing condition (Li & Rabitz, 2010) in its search for the optimum expansion coefficients. This method is described in Text S2 in Supporting Information S1.

**Figure 2:**
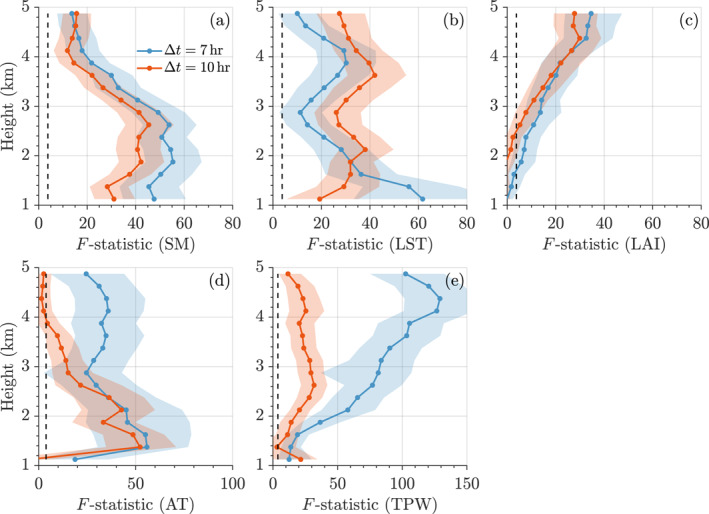
Vertical profiles of the mean F‐statistic of the first‐order component functions of panel (a) SM: $f_{1}\left(x_{1}\right)$, (b) LST: $f_{2}\left(x_{2}\right)$, (c) LAI: $f_{3}\left(x_{3}\right)$, (d) AT: $f_{4}\left(x_{4}\right)$, and (e) TPW: $f_{5}\left(x_{5}\right)$ computed from 1,000 bootstrap trials. Solid blue and red lines differentiate between temporal lags (Δt=7 and 10 hr) and black dashed lines represent the critical value $F_{\text{crit}}$ at confidence level $p_{\alpha }=0.95$. Values above $F_{\text{crit}}$ indicate the statistical significance of the relationship between the land‐surface/atmospheric variables and the cloud reflectivity. The light blue and light red regions portray the 95% bootstrap confidence intervals.

The statistical significance of a particular component function is readily determined by comparing the performance of the function expansion with and without this component function. Let ${\text{SSR}}_{1}$ be the sum of the squared residuals of the function $y=y_{0}+\sum_{i=1}^{d-1}f_{i}\left(x_{i}\right)$ with $l_{1}=\left(d-1\right)p$ expansion coefficients and SSR is the same quantity for the function $y=y_{0}+\sum_{i=1}^{d}f_{i}\left(x_{i}\right)$ expanded with $f_{d}\left(x_{d}\right)$ and $l=l_{1}+p$ coefficients. To reject the null hypothesis, “$H_{0}:f_{d}\left(x_{d}\right)\;\text{is}\;\text{insignificant}$”, the F‐statistic

(4) $$F=\frac{\left({\text{SSR}}_{1}-\text{SSR}\right)/\left(l-l_{1}\right)}{{\text{SSR}}_{1}/\left(n-l_{1}\right)},$$

must exceed $F_{\text{crit}}=F_{F}^{-1}\left(1-\alpha |l_{1}-l,n-l_{1}\right)$ where $F_{F}^{-1}\left(p_{\alpha }|\nu_{1},\nu_{2}\right)$ is the quantile function of the Fisher‐Snedecor distribution with $\nu_{1}$ and $\nu_{2}$ degrees of freedom at $p_{\alpha }=1-\alpha$ and significance level α∈(0,1). The magnitude of the F‐statistic conveys the importance of $f_{d}\left(x_{d}\right)$ in explaining the CVP and is, thus, a measure of the feedback strength. Here, we are mainly interested in $f_{1}\left(x_{1}\right),\text{…},f_{d}\left(x_{d}\right)$, as these first‐order component functions quantify the direct contribution of $x_{1},\text{…},x_{d}$ to the CVP (shown in next section). Most of the second‐order component functions, except for $f_{23}\left(x_{2},x_{3}\right)$ and $f_{34}\left(x_{3},x_{4}\right)$, contribute substantially less to the CVP, with F‐statistics (see Figure S4 in Supporting Information S1) that are almost an order of magnitude smaller than their first‐order counterparts (see Figure 2). Despite this apparent insignificance, the second‐order component functions still exert control on $f_{1}\left(x_{1}\right),\text{…},f_{d}\left(x_{d}\right)$ through their shared use of basis functions. The extended bases guarantee hierarchical orthogonality.

We are left with the selection of auxiliary land‐surface and atmospheric variables $\left(x_{2},\text{…},x_{d}\right)$ which complement SM, $x_{1}$, in explaining DPR‐measured cloud reflectivities, y. We settled on LST, LAI, AT, and TPW and, hence, $x={\left(\text{SM},\text{LST},\text{LAI},\text{AT},\text{TPW}\right)}^{⊤}$ is a 5×1 vector. LST and LAI modulate evapotranspiration under SM‐limited or energy‐limited regimes (Seneviratne et al., 2010) and AT and TPW contain synoptic information for the SMCPF about atmospheric preconditioning (Ford, Quiring, et al., 2015; Tuttle & Salvucci, 2017). Figure S1 in Supporting Information S1 presents a correlogram of the five input variables. The decision which input variables to use in regression analysis is always somewhat arbitrary. Our five input variables measure different and complementary properties of the land‐surface and overlying air column. One advantage of using ERA‐5 reanalysis data of AT and TPW in the HDMR decomposition is that we account explicitly for the dependence of the CVP on the coarse‐scale atmospheric conditions (Tuttle & Salvucci, 2017). The F‐statistic in Equation 4 will then determine the additional direct contribution of local land surface conditions to the CVP beyond what can be explained by synoptic conditions. Hence, the use of AT and TPW should enhance our confidence in the inferred causal relationships between local SM and the CVP. We do not consider the latent heat flux (LHF) in our HDMR decomposition. The LHF is a result of model simulation and controlled by several of our HDMR inputs. Any assumed constitutive relationship between SM, LST and LAI and the LHF will trouble inference of the individual and cooperative effects of these land surface variables on the CVP. Indeed, LHF inclusion substantially reduces the magnitude of the direct contribution of SM and LST to the CVP (not shown).

## 4 Results

### 4.1 Cloud Height and Temporal Lag of SMCPF

Figure 2 displays the F‐statistics of the (a) SM, (b) LST, (c) LAI, (d) AT, and (e) TPW component functions as a function of cloud height (1–5 km) and time lag (Δt=7 and 10 hr). The control that land‐surface and atmospheric variables exert on the CVP varies with cloud height. In the case of SM in panel (a) this equates to a height‐dependent SMCPF with a bottom‐heavy relationship between SM and CVP, at about 1–3 km above the surface. Above this level, the impact of SM on the CVP decreases rapidly with altitude. As we will show in Section 4.2, the first‐order SM component function $f_{1}\left(x_{1}\right)$ displays a positive feedback due to a wet soil. A higher SM implies a larger evaporative fraction, promoting moderate PBL growth (see Figure S2 in Supporting Information S1) and moisture accumulation (Yin et al., 2015). The CVP at higher altitudes is less dependent on surface SM and controlled more by the upper atmosphere at levels of 3 km and above (Findell & Eltahir, 2003a). Furthermore, a capping inversion layer can inhibit the upward motion of warm, moist air from the surface to the free atmosphere (Findell & Eltahir, 2003b). Indeed, the HDMR‐inferred relationship between SM and CVP as articulated by the F‐statistic is corroborated by simulation analyses (Findell & Eltahir, 2003a; Koukoula et al., 2019). The strong agreement in the results of the two time lags is a result of SM autocorrelation. The Δt=7 hour time lag displays the largest influence on the CVP at all measurement heights.

While the impact of SM on the CVP is most pronounced near the surface, LST affects the CVP over a much larger range of altitudes (see Figure 2b). Its F‐statistic displays a bimodal relationship with height, peaking close to the surface for Δt=7 h and at much higher altitudes of about 3.5–4.0 km for Δt=10 h. As will be further discussed in the next section, $f_{2}\left(x_{2}\right)$ exhibits a positive correlation with LST, suggesting that positive LST anomalies (dry soils) play an important role in shaping the CVP. The finding that the lower level CVP (1.0–2.5 km) is controlled by LST is in qualitative agreement with the pathway of negative SMCPFs. Positive LST anomalies enhance the sensible heat flux and convective triggering potential (CTP), thereby promoting rapid PBL growth. Figure S2 in Supporting Information S1 testifies to this conjecture and displays PBL heights from ERA‐5 reanalysis data. The response of the PBL height to wet and dry surfaces is in agreement with results from simulation‐based and observational studies (Findell & Eltahir, 2003a; Ford et al., 2023; Xu et al., 2021), which suggest two mechanisms for convective initiation: significant moistening of the PBL (over wet soil) and rapid growth of the PBL (over dry soil). Note that the LST contribution to the CVP decreases at 3.0 km height to increase again between 3.5 and 4.0 km. The reason why LST is influential at higher altitudes may be twofold. On the one hand, LST anomalies favor a strong CTP, increasing the capability of air parcels to overcome convective inhibition and reach the level of free convection (Taylor et al., 2012). If $f_{2}\left(x_{2}\right)$ measures the contribution of near‐surface air to cloud reflectivity for a specific height and time lag, then its F‐statistic (in Figure 2b) somehow approximates the dynamics of convective updrafts such that the largest F‐statistic value shifts from Δt=7 hours to Δt=10 hours with height changing from 1.0 to 5.0 km. Local LST may also influence higher‐altitude CVP by elevating the altitude of the melting layer, which is characterized by a sharp increase in radar reflectivity and resides between 3.0 and 5.0 km above the surface during the pre‐monsoon and monsoon seasons in the central United States (Song et al., 2021).

The F‐statistic of the LAI component, $f_{3}\left(x_{3}\right)$, has a negligible impact on the CVP in the lower atmosphere, due to the governing effects of SM, LST, and AT (see Figure 2d) on initiating convection and the subsequent formation of clouds and precipitation. LAI has a somehow stronger impact on the higher‐level CVP. The $f_{3}\left(x_{3}\right)$ component function in Figure S3a in Supporting Information S1 suggests that dense vegetation tends to suppress cloud formation in the upper atmosphere at 3–5 km height. A possible explanation is the role of LAI in modulating surface energy partitioning (Lauwaet et al., 2009), with denser vegetation potentially reducing surface heating and constraining the development of deep convection. Text S3 in Supporting Information S1 presents an analysis of these atmospheric controls on the CVP and physical underpinning comparable to that of the land‐surface variables.

The second‐order component functions have only a small contribution to the CVP with exceptions of the LST‐LAI component, $f_{23}\left(x_{2},x_{3}\right)$, and the LAI‐AT component, $f_{34}\left(x_{3},x_{4}\right)$. The joint contributions of LST‐LAI and LAI‐AT to the CVP at 1–3 km height may be linked to processes such as plant transpiration and boundary layer moistening. This is further illustrated in Figure S4 in Supporting Information S1 and discussed in Text S4 in Supporting Information S1. The comparison between HDMR and traditional data‐driven methods (see Figures S5–S6 and Text S5 in Supporting Information S1) reveals that all three approaches offer valuable insights into the diurnal SMCPF. However, HDMR is able to discern time lags in this feedback and captures the evolving physical dynamics of thermal updrafts.

### 4.2 The SMCPF Across Space

In this section, we focus on the spatial pattern of the SMCPF within the study region. We reiterate that we conduct functional decomposition of the cloud reflectivity using all the samples of April–October (2016–2019) for a specific time lag and cloud height, to guarantee an adequate number of samples and storm events. Our goal here is to present a 4‐year averaged spatial distribution of the derived component functions and identify locations of positive and negative SMCPF rather than focusing on interannual and/or cross‐season variations.

**Figure 3:**
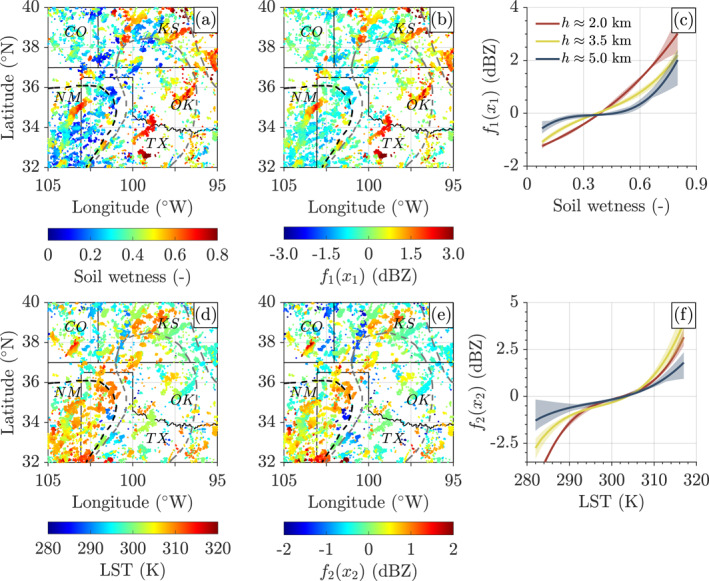
The central United States (95°W−105°W, 32°N−40°N) with (a) antecedent 7‐hr SMAP/L4 soil wetness (‐) collocated at coordinates of the GPM/DPR/L2A samples of April–October (2016–2019) and (b) first‐order component function of soil wetness, $f_{1}\left(x_{1}\right)$ (dBZ), evaluated at approximately 2.0 km height. Solid black lines delineate the state borders while dashed black and gray lines depict the negative feedback and transitional regions proposed by Findell and Eltahir (2003b). Panel (c) displays $f_{1}\left(x_{1}\right)$ (dBZ) as a function of antecedent 7‐hr SM. Values of $f_{1}\left(x_{1}\right)$ are evaluated at three separate heights: 2.0 km (red), 3.5 km (yellow), and 5.0 km (blue). The light‐colored regions correspond to the 95% bootstrap confidence intervals. The bottom row of panels presents the same content as panels (a–c) but for panel (d) SMAP/L4 LST and (e, f) its associated component function, $f_{2}\left(x_{2}\right)$.

Figures 3a and 3b present the spatial distribution of the antecedent 7‐hr SMAP/L4 soil wetness at the top layer (0–5 cm), collocated at the coordinates of the GPM/DPR/L2A samples of April–October (2016–2019), alongside the corresponding first‐order component function, $f_{1}\left(x_{1}\right)$ (dBZ), evaluated at 2.0 km. The GPM/DPR/L2A samples are spatio‐temporally scattered, directly corresponding to the satellite's path and observation times. This results in voided areas on the map. Our examination of SM's feedback strength, conditioned on an altitude of 2.0 km and a 7‐hr time lag, is of particular interest upon our prior analysis of the F‐statistic in Figure 2a. Panels (b–c) reveal the positive feedback from SM represented by $f_{1}\left(x_{1}\right)$. With a degree of saturation exceeding 0.4, wet soil could increase cloud reflectivity by up to 4 dBZ. The fact that the absolute value of $f_{1}\left(x_{1}\right)$ decreases with height in Panel (c) again lends support to our inferred height‐dependent SMCPF in Section 4.1, underscoring the stronger coupling between SM and CVP in the low‐level atmosphere. As a byproduct, we demonstrate in Text S6 and Figure S7 in Supporting Information S1 the application of the Marshall and Palmer (1948) formula to transform $f_{1}\left(x_{1}\right)$ (dBZ) into estimates of rainfall rate.

Significant positive feedback of SM is evident in regions such as northern Texas, central Oklahoma, northwestern and southeastern Kansas, and northeastern New Mexico. All these areas, with the exception of northeastern New Mexico, are located inside or close to the ’transitional regions' as delineated by the dashed gray lines as categorized by Findell and Eltahir (2003b). The middle transitional region, spanning from the semi‐arid southwestern to the humid southeastern parts of the central United States, is influenced by both the dry and wet soil advantage regimes (Findell & Eltahir, 2003b). Hence, this dual influence explicates the observable positive feedback in the central and eastern sections of the transitional region and negative feedback in the southwestern part (detailed below). These local wet soil anomalies can be attributed to early warm‐season mesoscale convective systems (MCSs) (Hu et al., 2021).

Since SM can indirectly exert feedback on cloud and precipitation through heating or cooling the surface (Duerinck et al., 2016), we further delve into examining spatially the samples of antecedent 7‐hr LST (K) and their contribution to cloud, $f_{2}\left(x_{2}\right)$ (dBZ), and rainfall, ΔR (mm/hour), in Figures 3d–3f and Figure S7 in Supporting Information S1, respectively. $f_{2}\left(x_{2}\right)$ exhibits a non‐linear dependence on LST where LST anomalies exert the most significant influence. From Figures 3d and 3e, it is suggested that LST above 305 K accounts for an increase of up to 4.0 dBZ in the cloud reflectivity and 2.0 mm/hr in rainfall rate (see Figure S7 in Supporting Information S1) at both 2.0 and 3.5 km. On the contrary, the samples with a cooler surface (LST<290 K) seem to foster a more stable atmospheric state, thereby reducing cloud reflectivity, especially in the lower troposphere (h≈2.0 km).

Geographically, the most significant effects of these anomalies are evident and clustered in the southwest of the study region (101°W−105°W and 32°N−36°N). Within this area, we find a significant negative correlation (R=−0.41, p<0.0001, shown in Figure S8a in Supporting Information S1) between surface SM and the LST component function, $f_{2}\left(x_{2}\right)$. Moreover, we illustrate in Figure S8b in Supporting Information S1 that LST contributes to CVP preferentially over dry soil with saturation between 0.1 and 0.4. These findings underscore the presence of the intrinsic SM‐LST coupling within SMCPF pathways (Seneviratne et al., 2010), and we can conveniently interpret $f_{2}\left(x_{2}\right)$ as a proxy for the indirect and negative feedback of SM on CVP. Notably, our identified negative feedback region (101°W−105°W, 32°N−36°N) is consistent with the one proposed by Findell and Eltahir (2003b) (represented by the black dashed line in Figures 3d and 3e). Several factors can play a role when it comes to the sources of convective clouds and precipitation over the dry soil. For instance, the monsoonal moisture incursion into New Mexico can increase local humidity and offset the reduced evapotranspiration from the local dry soils (Klein & Taylor, 2020; Wallace et al., 1999). Besides, the Great Plains Low‐Level Jet (GPLLJ) can transport abundant moisture southerly from the Gulf of Mexico into the central United States (Feng et al., 2016; Ford, Rapp, & Quiring, 2015).

## 5 Discussion and Conclusion

In this data‐driven study, we used functional decomposition of a large database of satellite‐measured soil moisture (SMAP/L4) and cloud vertical profiles (GPM/DPR/L2A) to quantify the relationship between topsoil moisture content (SM), land‐surface temperature (LST), leaf‐area index (LAI), atmospheric temperature (AT), total precipitable water (TPW) and cloud reflectivity in the central United States. Results show that the sign and strength of this soil moisture‐cloud‐precipitation feedback (SMCPF) differs substantially between cloud heights and geographical locations. A significant positive feedback is observed in the lower atmosphere, particularly between 1.0 and 3.0 km and SM time lag of 7 hr. Wet soils (saturation degree of 0.4 and larger) can increase the cloud reflectivity (rainfall rate) by up to 4.0 dBZ (2.0 mm/hr) at a height of about 2.0 km. This is most evident in northern Texas, central Oklahoma, and northwestern and southeastern Kansas. Negative SMCPF feedbacks, are a result of LST anomalies and extend from 1.0 to 4.0 km at a time lag of 7–10 hr. These LST anomalies display comparable increments in cloud reflectivity and rainfall rates to SM, except for northwestern Texas and southeastern and eastern New Mexico. The patterns of SMCPF identified by our satellite data decomposition method are in qualitative agreement with the findings of previous studies (Findell & Eltahir, 2003a, 2003b; Ford et al., 2023; Hu et al., 2021; Koukoula et al., 2019; Qian et al., 2013; Sathyanadh et al., 2017; Su & Dickinson, 2017).

We could have resorted to multivariate linear regression and written the DPR‐measured cloud reflectivities as a superposition of linear multiples of the input variables. This approach has elements in common with the multivariable function expansion of Soboľ. (1993) but by pooling together structural, cooperative and/or interaction contributions, linear regression may not accurately portray the direct effects of the input variables on the CVP. This should not discourage anyone from using traditional regression methods in hydroclimatological and hydrometeorological research. For example, G. Wang et al. (2024) uses linear regression to single out the influence of lower‐tropospheric moisture on the local SMCPF.

The ANOVA‐inspired functional decomposition of HDMR accurately disentangles the individual and correlative contributions of single and groups of correlated input variables to DPR‐measured cloud reflectivities. HDMR is a powerful addition to the toolbox of regression methods used by hydrometeorologists and hydroclimatologists for analyzing the complex and intricate relationships between land‐surface and atmospheric variables. HDMR results can help diagnose biases in the current generation of weather and climate models and help detect and quantify changes in SMCPFs at regional and global scales as a result of climate change and hydroclimatic extremes.

HDMR functional decomposition, however, requires a relatively large number of samples at each cloud height. This complicates seasonal, interannual, or localized analysis of the SMCPF. Another limitation of HDMR is that the number of input variables should not exceed, say, d=10, otherwise the matrices involved may become too large‐sized complicating the estimation of the component functions' expansion coefficients with D‐MORPH regression. Our current set of d=5 input variables serves as an illustration of the HDMR method but may ignore other variables that control the magnitude and sign of SMCPFs.

## Supporting information

Supporting Information S1

## Data Availability

The SMAP/L4 (L4_SM) product is obtained from the National Snow and Ice Data Center at Reichle et al. (2022). The GPM/DPR/L2A product (GPM_2ADPR) is obtained from the Goddard Earth Sciences Data and Information Services Center at GPM Science Team (2021). MATLAB processing scripts, along with the final data set of collocated SMAP and DPR samples, have been archived on Zenodo (Gao et al., 2024) with Creative Commons Attribution 4.0 International license.

## References

[grl68445-bib-0001] Baker, J. C. A. , Castilho de Souza, D. , Kubota, P. Y. , Buermann, W. , Coelho, C. A. S. , Andrews, M. B. , et al. (2021a). An assessment of land–atmosphere interactions over South America using satellites, reanalysis, and two global climate models. Journal of Hydrometeorology, 22(4), 905–922. 10.1175/JHM-D-20-0132.1

[grl68445-bib-0002] Baker, J. C. A. , Garcia‐Carreras, L. , Buermann, W. , De Souza, D. C. , Marsham, J. H. , Kubota, P. Y. , et al. (2021b). Robust Amazon precipitation projections in climate models that capture realistic land–atmosphere interactions. Environmental Research Letters, 16(7), 074002. 10.1088/1748-9326/abfb2e

[grl68445-bib-0003] Berg, A. , Findell, K. , Lintner, B. R. , Gentine, P. , & Kerr, C. (2013). Precipitation sensitivity to surface heat fluxes over North America in reanalysis and model data. Journal of Hydrometeorology, 14(3), 722–743. 10.1175/JHM-D-12-0111.1

[grl68445-bib-0004] Deardorff, J. W. (1980). Stratocumulus‐capped mixed layers derived from a three‐dimensional model. Boundary‐Layer Meteorology, 18(4), 495–527. 10.1007/BF00119502

[grl68445-bib-0005] Duerinck, H. M. , Van der Ent, R. J. , Van de Giesen, N. C. , Schoups, G. , Babovic, V. , & Yeh, P. J. F. (2016). Observed soil moisture–precipitation feedback in Illinois: A systematic analysis over different scales. Journal of Hydrometeorology, 17(6), 1645–1660. 10.1175/JHM-D-15-0032.1

[grl68445-bib-0006] Ek, M. B. , & Holtslag, A. A. M. (2004). Influence of soil moisture on boundary layer cloud development. Journal of Hydrometeorology, 5(1), 86–99. 10.1175/1525-7541(2004)005〈0086:IOSMOB〉2.0.CO;2

[grl68445-bib-0007] Falchi, A. , Minisci, E. , Kubicek, M. , Vasile, M. , & Lemmens, S. (2018). HDMR‐based sensitivity analysis and uncertainty quantification of GOCE aerodynamics using DSMC. Stardust Final Conference, 301–323. 10.1007/978-3-319-69956-1∖\text{\_}18

[grl68445-bib-0008] Fast, J. D. , Berg, L. K. , Feng, Z. , Mei, F. , Newsom, R. , Sakaguchi, K. , & Xiao, H. (2019). The impact of variable land‐atmosphere coupling on convective cloud populations observed during the 2016 HI‐SCALE field campaign. Journal of Advances in Modeling Earth Systems, 11(8), 2629–2654. 10.1029/2019MS001727

[grl68445-bib-0009] Feng, Z. , Leung, L. R. , Hagos, S. , Houze, R. A. , Burleyson, C. D. , & Balaguru, K. (2016). More frequent intense and long‐lived storms dominate the springtime trend in central US rainfall. Nature Communications, 7(1), 13429. 10.1038/ncomms13429 PMC5114602 27834368

[grl68445-bib-0010] Ferguson, C. R. , & Wood, E. F. (2011). Observed land‐atmosphere coupling from satellite remote sensing and reanalysis. Journal of Hydrometeorology, 12(6), 1221–1254. 10.1175/2011JHM1380.1

[grl68445-bib-0011] Findell, K. L. , & Eltahir, E. A. B. (2003a). Atmospheric controls on soil moisture–boundary layer interactions. Part I: Framework development. Journal of Hydrometeorology, 4(3), 552–569. 10.1175/1525-7541(2003)004〈0552:ACOSML〉2.0.CO;2

[grl68445-bib-0012] Findell, K. L. , & Eltahir, E. A. B. (2003b). Atmospheric controls on soil moisture–boundary layer interactions. Part II: Feedbacks within the continental United States. Journal of Hydrometeorology, 4(3), 570–583. 10.1175/1525-7541(2003)004〈0570:ACOSML〉2.0.CO;2

[grl68445-bib-0013] Findell, K. L. , Gentine, P. , Lintner, B. R. , & Kerr, C. (2011). Probability of afternoon precipitation in eastern United States and Mexico enhanced by high evaporation. Nature Geoscience, 4(7), 434–439. 10.1038/ngeo1174

[grl68445-bib-0014] Ford, T. W. , Quiring, S. M. , Frauenfeld, O. W. , & Rapp, A. D. (2015a). Synoptic conditions related to soil moisture‐atmosphere interactions and unorganized convection in Oklahoma. Journal of Geophysical Research: Atmospheres, 120(22), 11–519. 10.1002/2015jd023975

[grl68445-bib-0015] Ford, T. W. , Rapp, A. D. , & Quiring, S. M. (2015b). Does afternoon precipitation occur preferentially over dry or wet soils in Oklahoma? Journal of Hydrometeorology, 16(2), 874–888. 10.1175/JHM-D-14-0005.1

[grl68445-bib-0016] Ford, T. W. , Rapp, A. D. , Quiring, S. M. , & Blake, J. (2015c). Soil moisture–precipitation coupling: Observations from the Oklahoma Mesonet and underlying physical mechanisms. Hydrology and Earth System Sciences, 19(8), 3617–3631. 10.5194/hess-19-3617-2015

[grl68445-bib-0017] Ford, T. W. , Steiner, J. , Mason, B. , & Quiring, S. M. (2023). Observation‐driven characterization of soil moisture‐precipitation interactions in the Central United States. Journal of Geophysical Research: Atmospheres, 128(12), e2022JD037934. 10.1029/2022JD037934

[grl68445-bib-0018] Frye, J. D. , & Mote, T. L. (2010). Convection initiation along soil moisture boundaries in the southern Great Plains. Monthly Weather Review, 138(4), 1140–1151. 10.1175/2009MWR2865.1

[grl68445-bib-0019] Gao, Y. , Sahin, A. , & Vrugt, J. A. (2023). Probabilistic sensitivity analysis with dependent variables: Covariance‐based decomposition of hydrologic models. Water Resources Research, 59(4), e2022WR032834. 10.1029/2022WR032834

[grl68445-bib-0020] Gao, Y. , Vrugt, J. A. , Guilloteau, C. , & Foufoula‐Georgiou, E. (2024). Datasets and MATLAB scripts: Functional decomposition of satellite observations for studying soil moisture‐cloud‐precipitation feedback (version 1.0) [Software]. Zenodo. 10.5281/zenodo.13310556 PMC11582353 39582583

[grl68445-bib-0021] Gentine, P. , Holtslag, A. A. , D’Andrea, F. , & Ek, M. (2013). Surface and atmospheric controls on the onset of moist convection over land. Journal of Hydrometeorology, 14(5), 1443–1462. 10.1175/JHM-D-12-0137.1

[grl68445-bib-0022] Golaz, J. C. , Jiang, H. , & Cotton, W. R. (2001). A large‐eddy simulation study of cumulus clouds over land and sensitivity to soil moisture. Atmospheric Research, 59, 373–392. 10.1016/S0169-8095(01)00113-2

[grl68445-bib-0023] GPM Science Team . (2021). GPM DPR Ka environment L2A 1.5 hours 5 km V07 [Dataset]. NASA Goddard Earth Science Data and Information Services Center (GES DISC). Retrieved from https://disc.gsfc.nasa.gov/datacollection/GPM_2AKaENV_07.html

[grl68445-bib-0024] Graf, M. , Arnault, J. , Fersch, B. , & Kunstmann, H. (2021). Is the soil moisture precipitation feedback enhanced by heterogeneity and dry soils? A comparative study. Hydrological Processes, 35(9), e14332. 10.1002/hyp.14332

[grl68445-bib-0025] Guillod, B. P. , Orlowsky, B. , Miralles, D. , Teuling, A. J. , Blanken, P. D. , Buchmann, N. , et al. (2014). Land‐surface controls on afternoon precipitation diagnosed from observational data: Uncertainties and confounding factors. Atmospheric Chemistry and Physics, 14(16), 8343–8367. 10.5194/acp-14-8343-2014

[grl68445-bib-0026] Guillod, B. P. , Orlowsky, B. , Miralles, D. G. , Teuling, A. J. , & Seneviratne, S. I. (2015). Reconciling spatial and temporal soil moisture effects on afternoon rainfall. Nature Communications, 6(1), 6443. 10.1038/ncomms7443 PMC4366536 25740589

[grl68445-bib-0027] Han, C. , Brdar, S. , & Kollet, S. (2019). Response of convective boundary layer and shallow cumulus to soil moisture heterogeneity: A large‐eddy simulation study. Journal of Advances in Modeling Earth Systems, 11(12), 4305–4322. 10.1029/2019MS001772

[grl68445-bib-0028] Heinze, R. , Dipankar, A. , Henken, C. C. , Moseley, C. , Sourdeval, O. , Trömel, S. , et al. (2017). Large‐eddy simulations over Germany using ICON: A comprehensive evaluation. Quarterly Journal of the Royal Meteorological Society, 143(702), 69–100. 10.1002/qj.2947

[grl68445-bib-0029] Hohenegger, C. , Brockhaus, P. , Bretherton, C. S. , & Schär, C. (2009). The soil moisture‐precipitation feedback in simulations with explicit and parameterized convection. Journal of Climate, 22(19), 5003–5020. 10.1175/2009JCLI2604.1

[grl68445-bib-0030] Holloway, C. E. , & Neelin, J. D. (2010). Temporal relations of column water vapor and tropical precipitation. Journal of the Atmospheric Sciences, 67(4), 1091–1105. 10.1175/2009JAS3284.1

[grl68445-bib-0031] Hooker, G. (2007). Generalized functional ANOVA diagnostics for high‐dimensional functions of dependent variables. Journal of Computational & Graphical Statistics, 16(3), 709–732. 10.1198/106186007X237892

[grl68445-bib-0032] Hu, H. , Leung, L. R. , & Feng, Z. (2021). Early warm‐season mesoscale convective systems dominate soil moisture–precipitation feedback for summer rainfall in central United States. Proceedings of the National Academy of Sciences, 118(43), e2105260118. 10.1073/pnas.2105260118 PMC8639340 34663726

[grl68445-bib-0033] Iguchi, T. , Seto, S. , Meneghini, R. , Yoshida, N. , Awaka, J. , Le, M. , et al. (2010). GPM/DPR level‐2 algorithm theoretical basis document. NASA Goddard Space Flight Center.

[grl68445-bib-0034] Klein, C. , & Taylor, C. M. (2020). Dry soils can intensify mesoscale convective systems. Proceedings of the National Academy of Sciences, 117(35), 21132–21137. 10.1073/pnas.2007998117 PMC7474668 32817526

[grl68445-bib-0035] Knist, S. , Goergen, K. , Buonomo, E. , Christensen, O. B. , Colette, A. , Cardoso, R. M. , et al. (2017). Land‐atmosphere coupling in euro‐cordex evaluation experiments. Journal of Geophysical Research: Atmospheres, 122(1), 79–103. 10.1002/2016JD025476

[grl68445-bib-0036] Koster, R. D. , Dirmeyer, P. A. , Guo, Z. , Bonan, G. , Chan, E. , Cox, P. , et al. (2004). Regions of strong coupling between soil moisture and precipitation. Science, 305(5687), 1138–1140. 10.1126/science.1100217 15326351

[grl68445-bib-0037] Koster, R. D. , Liu, Q. , Mahanama, S. P. P. , & Reichle, R. H. (2018). Improved hydrological simulation using SMAP data: Relative impacts of model calibration and data assimilation. Journal of Hydrometeorology, 19(4), 727–741. 10.1175/JHM-D-17-0228.1 29983646 PMC6031932

[grl68445-bib-0038] Koster, R. D. , Sud, Y. C. , Guo, Z. , Dirmeyer, P. A. , Bonan, G. , Oleson, K. W. , et al. (2006). Glace: The global land–atmosphere coupling experiment. Part I: Overview. Journal of Hydrometeorology, 7(4), 590–610. 10.1175/JHM510.1

[grl68445-bib-0039] Koukoula, M. , Nikolopoulos, E. I. , Kushta, J. , Bartsotas, N. S. , Kallos, G. , & Anagnostou, E. N. (2019). A numerical sensitivity analysis of soil moisture feedback on convective precipitation. Journal of Hydrometeorology, 20(1), 23–44. 10.1175/JHM-D-18-0134.1

[grl68445-bib-0040] Krakauer, N. Y. , Cook, B. I. , & Puma, M. J. (2010). Contribution of soil moisture feedback to hydroclimatic variability. Hydrology and Earth System Sciences, 14(3), 505–520. 10.5194/hess-14-505-2010

[grl68445-bib-0041] Kucherenko, S. , Feil, B. , Shah, N. , & Mauntz, W. (2011). The identification of model effective dimensions using global sensitivity analysis. Reliability Engineering & System Safety, 96(4), 440–449. 10.1016/j.ress.2010.11.003

[grl68445-bib-0042] Lasser, M. , O, S. , & Foelsche, U. (2019). Evaluation of GPM‐DPR precipitation estimates with WegenerNet gauge data. Atmospheric Measurement Techniques, 12(9), 5055–5070. 10.5194/amt-12-5055-2019

[grl68445-bib-0043] Lauwaet, D. , van Lipzig, N. P. M. , & De Ridder, K. (2009). The effect of vegetation changes on precipitation and Mesoscale Convective Systems in the Sahel. Climate Dynamics, 33(4), 521–534. 10.1007/s00382-009-0539-2

[grl68445-bib-0044] Li, G. , & Rabitz, H. (2010). D‐MORPH regression: Application to modeling with unknown parameters more than observation data. Journal of Mathematical Chemistry, 48(4), 1010–1035. 10.1007/s10910-010-9722-2

[grl68445-bib-0045] Li, G. , & Rabitz, H. (2012). General formulation of HDMR component functions with independent and correlated variables. Journal of Mathematical Chemistry, 50(1), 99–130. 10.1007/s10910-011-9898-0

[grl68445-bib-0046] Liao, L. , & Meneghini, R. (2022). GPM DPR retrievals: Algorithm, evaluation, and validation. Remote Sensing, 14(4), 843. 10.3390/rs14040843

[grl68445-bib-0047] Marshall, J. , & Palmer, W. M. (1948). The distribution of raindrops with size. Journal of meteorology, 5(4), 165–166. 10.1175/1520-0469(1948)005<0165:tdorws>2.0.co;2

[grl68445-bib-0048] Miralles, A. J. , Diego, G. T. , van Heerwaarden, C. C. , & Vilà‐Guerau de Arellano, J. (2014). Mega‐heatwave temperatures due to combined soil desiccation and atmospheric heat accumulation. Nature Geoscience, 7(5), 345–349. 10.1038/ngeo2141

[grl68445-bib-0049] Oerter, E. , Slessarev, E. , Visser, A. , Min, K. , Kan, M. , McFarlane, K. J. , et al. (2021). Hydraulic redistribution by deeply rooted grasses and its ecohydrologic implications in the southern Great Plains of North America. Hydrological Processes, 35(9), e14366. 10.1002/hyp.14366

[grl68445-bib-0050] Pejcic, V. , Saavedra Garfias, P. , Mühlbauer, K. , Trömel, S. , & Simmer, C. (2020). Comparison between precipitation estimates of ground‐based weather radar composites and GPM’s DPR rainfall product over Germany. Meteorologische Zeitschrift, 29(6), 451–466. 10.1127/metz/2020/1039

[grl68445-bib-0051] Qian, Y. , Huang, M. , Yang, B. , & Berg, L. K. (2013). A modeling study of irrigation effects on surface fluxes and land–air–cloud interactions in the Southern Great Plains. Journal of Hydrometeorology, 14(3), 700–721. 10.1175/JHM-D-12-0134.1

[grl68445-bib-0052] Rabitz, H. , & Aliş, Ö. F. (1999). General foundations of high‐dimensional model representations. Journal of Mathematical Chemistry, 25(2), 197–233. 10.1023/A:1019188517934

[grl68445-bib-0053] Reichle, R. H. , De Lannoy, G. , Koster, R. D. , Crow, W. T. , Kimball, J. S. , & Liu, Q. (2022). SMAP L4 global 3‐hourly 9 km EASE‐Grid surface and root zone soil moisture analysis update, version 7 [Dataset]. NASA National Snow and Ice Data Center Distributed Active Archive Center. 10.5067/LWJ6TF5SZRG3

[grl68445-bib-0054] Reichle, R. H. , De Lannoy, G. J. M. , Liu, Q. , Koster, R. D. , Kimball, J. S. , Crow, W. T. , et al. (2017). Global assessment of the SMAP Level‐4 surface and root‐zone soil moisture product using assimilation diagnostics. Journal of Hydrometeorology, 18(12), 3217–3237. 10.1175/JHM-D-17-0130.1 30364509 PMC6196324

[grl68445-bib-0055] Reichle, R. H. , Lucchesi, R. A. , Ardizzone, J. V. , Kim, G.‐K. , Smith, E. B. , & Weiss, B. H. (2015). Soil moisture active passive (SMAP) mission level 4 surface and root zone soil moisture (L4_SM) product specification document (tech. rep.). NASA Goddard Space Flight Center. Retrieved from https://nsidc.org/sites/default/files/reichle789.pdf

[grl68445-bib-0056] Sakaguchi, K. , Berg, L. K. , Chen, J. , Fast, J. , Newsom, R. , Tai, S. , et al. (2022). Determining spatial scales of soil moisture—Cloud coupling pathways using semi‐idealized simulations. Journal of Geophysical Research: Atmospheres, 127(2), e2021JD035282. 10.1029/2021JD035282

[grl68445-bib-0057] Santanello, J. A. , Peters‐Lidard, C. D. , Kennedy, A. , & Kumar, S. V. (2013). Diagnosing the nature of land‐atmosphere coupling: A case study of dry/wet extremes in the us southern great plains. Journal of Hydrometeorology, 14(1), 3–24. 10.1175/JHM-D-12-023.1

[grl68445-bib-0058] Santanello, J. A. , Peters‐Lidard, C. D. , Kumar, S. V. , Alonge, C. , & Tao, W.‐K. (2009). A modeling and observational framework for diagnosing local land–atmosphere coupling on diurnal time scales. Journal of Hydrometeorology, 10(3), 577–599. 10.1175/2009JHM1066.1

[grl68445-bib-0059] Sathyanadh, A. , Prabha, T. V. , Balaji, B. , Resmi, E. A. , & Karipot, A. (2017). Evaluation of WRF PBL parameterization schemes against direct observations during a dry event over the Ganges valley. Atmospheric Research, 193, 125–141. 10.1016/j.atmosres.2017.02.016

[grl68445-bib-0060] Schär, C. , Lüthi, D. , Beyerle, U. , & Heise, E. (1999). The soil–precipitation feedback: A process study with a regional climate model. Journal of Climate, 12(3), 722–741. 10.1175/1520-0442(1999)012〈0722:TSPFAP〉2.0.CO;2

[grl68445-bib-0061] Schlemmer, L. , Hohenegger, C. , Schmidli, J. , & Schär, C. (2012). Diurnal equilibrium convection and land surface–atmosphere interactions in an idealized cloud‐resolving model. Quarterly Journal of the Royal Meteorological Society, 138(667), 1526–1539. 10.1002/qj.1892

[grl68445-bib-0062] Seneviratne, S. I. , Corti, T. , Davin, E. L. , Hirschi, M. , Jaeger, E. B. , Lehner, I. , et al. (2010). Investigating soil moisture–climate interactions in a changing climate: A review. Earth‐Science Reviews, 99(3), 125–161. 10.1016/j.earscirev.2010.02.004

[grl68445-bib-0063] Seneviratne, S. I. , Lüthi, D. , Litschi, M. , & Schär, C. (2006). Land–atmosphere coupling and climate change in europe. Nature, 443(7108), 205–209. 10.1038/nature05095 16971947

[grl68445-bib-0064] Shereena, O. A. , & Rao, B. N. (2019). HDMR‐based Bayesian structural system identification. In Recent advances in structural engineering (Vol. 1, pp. 453–464). Springer. 10.1007/978-981-13-0362-3_36

[grl68445-bib-0065] Sherwood, S. C. (1999). Convective precursors and predictability in the tropical western Pacific. Monthly Weather Review, 127(12), 2977–2991. 10.1175/1520-0493(1999)127〈2977:CPAPIT〉2.0.CO;2

[grl68445-bib-0066] Soboľ, I. M. (1993). Sensitivity estimates for nonlinear mathematical models. Mathematical Modelling in Civil Engineering, 1(4), 407–414.

[grl68445-bib-0067] Song, J. I. , Yum, S. S. , Park, S. H. , Kim, K. H. , Park, K. J. , & Joo, S. W. (2021). Climatology of melting layer heights estimated from cloud radar observations at various locations. Journal of Geophysical Research: Atmospheres, 126(17), e2021JD034816. 10.1029/2021JD034816

[grl68445-bib-0068] Spennemann, P. C. , Salvia, M. , Ruscica, R. C. , Sörensson, A. A. , Grings, F. , & Karszenbaum, H. (2018). Land‐atmosphere interaction patterns in southeastern South America using satellite products and climate models. International Journal of Applied Earth Observation and Geoinformation, 64, 96–103. 10.1016/j.jag.2017.08.016

[grl68445-bib-0069] Su, H. , & Dickinson, R. E. (2017). On the spatial gradient of soil moisture–precipitation feedback strength in the April 2011 drought in the Southern Great Plains. Journal of Climate, 30(3), 829–848. 10.1175/JCLI-D-13-00185.1

[grl68445-bib-0070] Tavakol, A. , Rahmani, V. , Quiring, S. M. , & Kumar, S. V. (2019). Evaluation analysis of NASA SMAP L3 and L4 and SPoRT‐LIS soil moisture data in the United States. Remote Sensing of Environment, 229, 234–246. 10.1016/j.rse.2019.05.006

[grl68445-bib-0071] Tawfik, A. B. , Dirmeyer, P. A. , & Santanello, J. A. (2015). The heated condensation framework.: Part i: Description and southern great plains case study. Journal of Hydrometeorology, 16(5), 1929–1945. 10.1175/jhm-d-14-0117.1

[grl68445-bib-0072] Taylor, C. M. (2015). Detecting soil moisture impacts on convective initiation in Europe. Geophysical Research Letters, 42(11), 4631–4638. 10.1002/2015GL064030

[grl68445-bib-0073] Taylor, C. M. , de Jeu, R. A. M. , Guichard, F. , Harris, P. P. , & Dorigo, W. A. (2012). Afternoon rain more likely over drier soils. Nature, 489(7416), 423–426. 10.1038/nature11377 22972193

[grl68445-bib-0074] Taylor, C. M. , & Ellis, R. J. (2006). Satellite detection of soil moisture impacts on convection at the mesoscale. Geophysical Research Letters, 33(3). 10.1029/2005GL025252

[grl68445-bib-0075] Taylor, C. M. , Gounou, A. , Guichard, F. , Harris, P. P. , Ellis, R. J. , Couvreux, F. , & De Kauwe, M. (2011). Frequency of Sahelian storm initiation enhanced over mesoscale soil‐moisture patterns. Nature Geoscience, 4(7), 430–433. 10.1038/ngeo1173

[grl68445-bib-0076] Taylor, C. M. , Harris, P. P. , & Parker, D. J. (2010). Impact of soil moisture on the development of a Sahelian mesoscale convective system: A case‐study from the AMMA special observing period. Quarterly Journal of the Royal Meteorological Society, 136(S1), 456–470. 10.1002/qj.465

[grl68445-bib-0077] Thompson, G. , Field, P. R. , Rasmussen, R. M. , & Hall, W. D. (2008). Explicit forecasts of winter precipitation using an improved bulk microphysics scheme. Part II: Implementation of a new snow parameterization. Monthly Weather Review, 136(12), 5095–5115. 10.1175/2008MWR2387.1

[grl68445-bib-0078] Thompson, G. , Rasmussen, R. M. , & Manning, K. (2004). Explicit forecasts of winter precipitation using an improved bulk microphysics scheme. Part I: Description and sensitivity analysis. Monthly Weather Review, 132(2), 519–542. 10.1175/1520-0493(2004)132〈0519:EFOWPU〉2.0.CO;2

[grl68445-bib-0079] Tuttle, S. E. , & Salvucci, G. D. (2017). Confounding factors in determining causal soil moisture‐precipitation feedback. Water Resources Research, 53(7), 5531–5544. 10.1002/2016WR019869

[grl68445-bib-0080] Wallace, C. E. , Maddox, R. A. , & Howard, K. W. (1999). Summertime convective storm environments in central Arizona: Local observations. Weather and Forecasting, 14(6), 994–1006. 10.1175/1520-0434(1999)014〈0994:SCSEIC〉2.0.CO;2

[grl68445-bib-0081] Wang, G. , Fu, R. , Zhuang, Y. , Dirmeyer, P. A. , Santanello, J. A. , Wang, G. , et al. (2024). Influence of lower‐tropospheric moisture on local soil moisture–precipitation feedback over the US Southern Great Plains. Atmospheric Chemistry and Physics, 24(6), 3857–3868. 10.5194/acp-24-3857-2024

[grl68445-bib-0082] Wang, G. , Kim, Y. , & Wang, D. (2007). Quantifying the strength of soil moisture–precipitation coupling and its sensitivity to changes in surface water budget. Journal of Hydrometeorology, 8(3), 551–570. 10.1175/JHM573.1

[grl68445-bib-0083] Wang, H. , Chen, L. , Ye, F. , & Chen, L. (2017). Global sensitivity analysis for fiber reinforced composite fiber path based on D‐MORPH‐HDMR algorithm. Structural and Multidisciplinary Optimization, 56(3), 697–712. 10.1007/s00158-017-1681-9

[grl68445-bib-0084] Welty, J. , & Zeng, X. (2018). Does soil moisture affect warm season precipitation over the southern Great Plains? Geophysical Research Letters, 45(15), 7866–7873. 10.1029/2018GL078598

[grl68445-bib-0085] Williams, I. N. (2019). Evaluating soil moisture feedback on convective triggering: Roles of convective and land‐model parameterizations. Journal of Geophysical Research: Atmospheres, 124(1), 317–332. 10.1029/2018JD029326

[grl68445-bib-0086] Xu, Z. , Chen, H. , Guo, J. , & Zhang, W. (2021). Contrasting effect of soil moisture on the daytime boundary layer under different thermodynamic conditions in summer over China. Geophysical Research Letters, 48(3), e2020GL090989. 10.1029/2020GL090989

[grl68445-bib-0087] Yin, J. , Albertson, J. D. , Rigby, J. R. , & Porporato, A. (2015). Land and atmospheric controls on initiation and intensity of moist convection: CAPE dynamics and LCL crossings. Water Resources Research, 51(10), 8476–8493. 10.1002/2015WR017286

[grl68445-bib-0088] Yin, J. , Porporato, A. , & Albertson, J. (2014). Interplay of climate seasonality and soil moisture‐rainfall feedback. Water Resources Research, 50(7), 6053–6066. 10.1002/2013WR014772

[grl68445-bib-0089] Yuan, S. , Wang, Y. , Quiring, S. M. , Ford, T. W. , & Houston, A. L. (2020). A sensitivity study on the response of convection initiation to in situ soil moisture in the central United States. Climate Dynamics, 54(3–4), 2013–2028. 10.1007/s00382-019-05098-0

[grl68445-bib-0090] Zhang, J. , Howard, K. , Langston, C. , Kaney, B. , Qi, Y. , Tang, L. , et al. (2016). Multi‐Radar Multi‐Sensor (MRMS) quantitative precipitation estimation: Initial operating capabilities. Bulletin of the American Meteorological Society, 97(4), 621–638. 10.1175/BAMS-D-14-00174.1

[grl68445-bib-0091] Zhang, L. , He, C. , & Zhang, M. (2017a). Multi‐scale evaluation of the SMAP product using sparse in‐situ network over a high mountainous watershed, Northwest China. Remote Sensing, 9(11), 1111. 10.3390/rs9111111

[grl68445-bib-0092] Zhang, X. , Zhang, T. , Zhou, P. , Shao, Y. , & Gao, S. (2017b). Validation analysis of SMAP and AMSR2 soil moisture products over the United States using ground‐based measurements. Remote Sensing, 9(2), 104. 10.3390/rs9020104

