# Supplementary material for: Soil Moisture‐Cloud‐Precipitation Feedback in the Lower Atmosphere From Functional Decomposition of Satellite Observations
Source: Geophys Res Lett. 2024 Nov 21;51(22):e2024GL110347. doi: 10.1029/2024GL110347 (PMC11582353; doi:10.1029/2024GL110347)
Supplement: Supplementary file 1 — Supporting Information S1 [file GRL-51-0-s001.pdf]

Supporting Information for "Soil Moisture-Cloud-Precipitation Feedback in the Lower Atmosphere from Functional Decomposition of Satellite Observations"

Yifu Gao[1], Clément Guilloteau[1], Efi Foufoula-Georgiou[1], Chonggang Xu[2],

Xiaoming Sun[2] and Jasper A. Vrugt[1] *

[1] Department of Civil and Environmental Engineering, University of California, Irvine, California, USA.

[2] Earth and Environmental Sciences Division, Los Alamos National Laboratory, Los Alamos, New Mexico, USA.

Contents of this file

1. Introduction
2. Texts S1 to S6
3. Figures S1 to S8

Introduction

This supporting information contains the following content: (1) Text S1: A more detailed description of the SMAP/L4 and GPM/DPR/L2A products; (2) Text S2: Component

*Corresponding author: jasper@uci.edu

function construction and D-MORPH regression that enforces hierarchical orthogonality of the component functions in pursuit of the optimum coefficients; (3) Text S3: Investigation into atmospheric controls on cloud vertical profile (CVP); (4) Text S4: Discussion of the F-statistic profiles of second-order component functions; (5) Text S5: Discussion of the results of correlation analysis and linear regression using the same cloud, land-surface, and atmospheric data as the main text; (6) Text S6: Application of Marshall-Palmer formula to converting component function, $f_i(x_i)$, into rainfall rate estimates; (7) Figure S1: Correlogram of the land-surface and atmospheric variables employed as inputs in the High-Dimensional Model Representation (HDMR): soil moisture (SM), land-surface temperature (LST), leaf area index (LAI), atmospheric temperature (AT), and total precipitable water (TPW); (8) Figure S2: Diurnal development of ERA5 reanalysis planetary boundary layer (PBL) height determined for two groups of samples where Groups 1 and 2 highlight positive and negative soil moisture cloud precipitation feedback (SMCPF), respectively; (9) Figure S3: Antecedent 7-hour LAI (-), AT (K), and TPW (kg/m^2) against their first-order component functions, $f_3(x_3)$, $f_4(x_4)$, and $f_5(x_5)$, respectively, evaluated at three separate heights; (10) Figure S4: Vertical profiles of the mean F-statistic of the second-order component functions; (11) Figure S5: Vertical profiles of Pearson correlation coefficient between cloud reflectivity and each land-surface or atmospheric variable; (12) Figure S6: Vertical profiles of coefficients $\widehat{\beta}_1, \ldots, \widehat{\beta}_5$ estimated for the multiple linear regression function, $y = f(\mathbf{x}, \boldsymbol{\beta}) = \beta_0 + \beta_1 x_1 + \ldots + \beta_5 x_5$; (13) Figure S7: same as Figure 3 of the main text but for the feedback of SM and LST on rainfall rate, ΔR (mm/hour); (14)

Figure S8: Evidence of negative SMCPF in the southwest (101°W-105°W, 32°N-36°N) of the central United States.

Text S1. Data Description: SMAP/L4 and DPR/L2A Products

The SMAP mission Level 4 SM (L4_SM) product provides 3-hourly estimates of surface and root-zone SM at 9-km spatial resolution with global coverage (Reichle et al., 2015). Despite the malfunction of SMAP's active radar system since July 2015, its passive microwave radiometer has continued to operate and measure brightness temperatures. SMAP L-band (1.4 GHz) brightness temperature data from descending and ascending half-orbit satellite passes (approximately 6:00 AM and 6:00 PM local solar time, respectively) are assimilated into the NASA catchment land-surface model using the Earth-fixed, global, cylindrical 9 km Equal-Area Scalable Earth Grid, Version 2.0 (EASE-Grid 2.0) projection. L4_SM provides surface (see Figure 1a) and root zone SM data in two products. We use the 3-hour time-averaged 9-km geophysical data product (SPL4SMGP). which provides soil wetness (0-1) of the top layer (0-5 cm) and other land-surface variables.

Cloud vertical profiles are derived from the dual-frequency precipitation radar (DPR) aboard the Global Precipitation Measurement (GPM) Core Observatory satellite. Launched in February 2014, the GPM core satellite orbits the Earth about 16 times a day in a non-sun-synchronous orbit with an inclination angle of 65°. The DPR operates at Ku-band (13.6 GHz) and Ka-band (35.5 GHz) frequencies and is an advanced successor to the Tropical Rainfall Measuring Mission precipitation radar. The DPR has the capability of obtaining the raindrop size distribution with improved detection of light rain and precipitating snow due to the addition of the Ka-band radar. This instrument operates in two modes: (1) a higher range resolution, lower sensitivity mode in the inner swath (125

km) and (2) a lower resolution, higher sensitivity mode (Liao & Meneghini, 2022). The KuPR and KaPR sense rain over land and ocean, day and night.

The GPM/DPR/L2A product (GPM_2ADPR) provides a swath of precipitation profiles (see Figure 1b) every 1.5 hours with a spatial resolution of 5 km and vertical increment of 125 m. Each pixel has its own cloud and precipitation profiles such as the cloud reflectivity factor (see Figure 1c), precipitation rate, height of received echos, and so forth. The DPR level-2 algorithm is made up of six different modules named preparation (PRE), vertical profile (VER), classification (CSF), drop size distribution (DSD), surface reference technique (SRT) and solver (SLV) (Iguchi et al., 2010). The SLV module computes the DSD, precipitation rate and related physical quantities by solving the radar equations recursively along range profiles utilizing output received from other modules such as the measured reflectivity profile (PRE), precipitation type (CSF), path integrated attenuation (SRT) and an adjustable $R - D_{\mathrm{m}}$ relationship of precipitation rate R and mass-weighted diameter D_{m} (DSD). We use the major data fields, zFactorFinal (dBZ) and typePrecip (-), which provide vertical profiles of the Ka-band cloud reflectivity factor and an 8-digit ID for precipitation type, respectively. In this study, we take the 250-m average Ka-band cloud reflectivity and exclusively use samples classified as convective precipitation.

Text S2. Component Function Construction and D-MORPH Regression

We construct the component functions using the family of orthogonal polynomial functions (Li & Rabitz, 2012)

$$\begin{array}{ccc} \phi_1(x_i) = a_1 x_i + a_0 & \phi_2(x_i) = b_2 x_i^2 + b_1 x_1 + b_0 & \phi_3(x_i) = c_3 x_i^3 + c_2 x_i^2 + c_1 x_i + c_0 \\ \text{degree } p = 1 & \text{degree } p = 2 & \text{degree } p = 3, \end{array} \tag{S1}$$

where the values of coefficients a, b and c are derived from Gram-Schmidt orthonormalization. This projection operator constructs an orthonormal basis for the polynomial functions on the unit interval of x with respect to an arbitrary weighting function. The component functions are now equal to sums of linear multiples of the orthonormalized polynomial functions of degrees 1 to p

$$f_i(x_i) = \sum_{r=1}^{p} \alpha_r^{(i)i} \phi_r(x_i) \tag{S2a}$$

$$f_{ij}(x_i, x_j) = \sum_{r=1}^{p} \left[\alpha_r^{(ij)i} \phi_r(x_i) + \alpha_r^{(ij)j} \phi_r(x_j) \right] + \sum_{r=1}^{p} \sum_{s=1}^{p} \beta_{rs}^{(ij)ij} \phi_r(x_i) \phi_s(x_j), \tag{S2b}$$

where the extended bases of the second-order component functions will help satisfy the vanishing condition in Equation (3). The use of extended bases has implications for our index notation of the coefficients. Parenthesized symbol(s) in the superscripts of α, β and γ enumerate the component functions. Non-parenthesized superscripts are indices of the input vector, $\mathbf{x}$. If all n_{12} component functions are included in the series expansion of equation (1) then the number of unknown expansion coefficients equals $l = dp + \frac{1}{2}d(d-1)(2p+p^2)$. At the end of Section 3, we introduce the five ($d = 5$) input variables used in our analysis. Thus, with a typical polynomial degree $p = 3$ (Gao et al., 2023) the number of unknown expansion coefficients $l = 165$ is much smaller than the sample size n for each cloud height (Figure 1d). This minimizes the risk of overfitting.

Hierarchical representation of the cloud reflectivity into a finite sum of first- and second-order polynomial component functions offers a significant advantage over function approximation methods such as artificial neural networks. The function expansion delineates marginal and cooperative effects in determining the magnitude and sign of the SMCPF. Furthermore, the expansion coefficients α, β and γ of the component functions of equation (S2) have a closed-form solution for a training record of $(\mathbf{x}, y)$-samples.

We can write equation (2) in matrix form $\boldsymbol{\Phi}\mathbf{c} = \mathbf{b}$ and yield

$$\boldsymbol{\Phi} = \begin{bmatrix} \boldsymbol{\phi}(\mathbf{x}^{(1)})^\top \\ \vdots \\ \boldsymbol{\phi}(\mathbf{x}^{(N)})^\top \end{bmatrix} \quad \text{and} \quad \mathbf{b} = \begin{bmatrix} y^{(1)} - y_0 \\ \vdots \\ y^{(N)} - y_0 \end{bmatrix}, \tag{S3a}$$

where $\boldsymbol{\phi}(\mathbf{x})^\top$ is a $1 \times l$ design vector with orthonormalized polynomial functions of equation (S2) (and products thereof) evaluated at their respective entries of $\mathbf{x}$ and arranged in appropriate order, $\mathbf{c}$ is a $l \times 1$ coefficient vector with values of α, β and γ and the $n \times 1$ vector $\mathbf{b}$ stores differences between the measured $y^{(i)}$ and mean y_0 cloud reflectivity for each training sample, $i = (1, \ldots, n)$. To offer some protection against underdetermined problems $N < l$ or a rank-deficient design matrix, we remove duplicate entries of the basis functions of the first- and second-order component functions. This reduced system is easier to solve in practice (Li & Rabitz, 2012). First, we determine the least squares values $\widehat{\mathbf{c}}_{\text{ls}}$ of the expansion coefficients

$$\widehat{\mathbf{c}}_{\text{ls}} = (\boldsymbol{\Phi}^\top \boldsymbol{\Phi})^\dagger \mathbf{d}, \tag{S4}$$

where the $l \times (l - dp)$ matrix $(\boldsymbol{\Phi}^\top \boldsymbol{\Phi})^\dagger$ is the generalized pseudo inverse of the $l \times l$ Gramian matrix, $\mathbf{G} = \boldsymbol{\Phi}^\top \boldsymbol{\Phi}$, which satisfies all four Moore-Penrose conditions (Penrose, 1955; Golub & Van Loan, 1996) and whose redundant rows (first dp rows of the first-order basis functions) are removed and $\mathbf{d}$ is the $(l - dp) \times 1$ vector $\boldsymbol{\Phi}^\top \mathbf{b}$ without the

first dp rows. Diffeomorphic modulation under observable response preserving homotopy (D-MORPH) regression (Li & Rabitz, 2010) enforces hierarchical orthogonality of the component functions in pursuit of the optimum coefficients

$$\widehat{\mathbf{c}}_{\mathrm{dm}} = \mathbf{V}_{l-r}(\mathbf{U}_{l-r}^{\top}\mathbf{V}_{l-r})\mathbf{U}_{l-r}^{\top}\widehat{\mathbf{c}}_{\mathrm{ls}}, \tag{S5}$$

where $\mathbf{U}_{l-r}$ and $\mathbf{V}_{l-r}$ equal the last $l-r$ columns of the $l \times l$ matrices $\mathbf{U}$ and $\mathbf{V}$ determined from singular value decomposition $\mathbf{PB} = \mathbf{U\Sigma V}^{\top}$ of the product of a $l \times l$ projection matrix $\mathbf{P} = \mathbf{I}_l - \mathbf{G}$ and $l \times l$ constraint matrix $\mathbf{B}$ of inner products of the orthonormalized polynomial functions. This latter matrix $\mathbf{B}$ enforces the relaxed vanishing condition in Equation (3) (Li & Rabitz, 2010), matrix $\mathbf{I}_l$ is the $l \times l$ identity matrix and r is the number of nonzero singular values.

Text S3. Atmospheric Controls on CVP

Figure 3d-e displays the similar content as panels (a-c) but for the two atmospheric conditions: low-level (roughly 1-3 km) AT and TPW, whose component functions are $f_4(x_4)$ and $f_5(x_5)$, respectively. As anticipated, antecedent 7-10 hours low-level AT significantly influences the cloud vertical profile (CVP) within the 1-3 km range, denoting a bottom-heavy relationship. The component function, $f_4(x_4)$, shows a strong negative correlation with AT (as illustrated in Figure S3b) and thus underscores the profound contribution of a cooler early atmosphere to the development of convective clouds/precipitation. The observed sensitivity of CVP to early-stage AT is deemed reasonable since AT is a crucial atmospherically forced synoptic condition for diagnosing the likelihood of deep convection. Conditions of lower AT coupled with higher LST are conducive to higher Convective Available Potential Energy (CAPE) and Convective Triggering Potential (CTP) (Findell & Eltahir, 2003a). Compared to TPW, AT exhibits weaker predictability in the free atmosphere, likely due to TPW's more straightforward connection with the cloud formation (as detailed below). Through integrating the characterized relationships between CVP and $\{\mathrm{SM}, \mathrm{LST}, \mathrm{AT}\}$ (i.e., $f_1(x_1)$, $f_2(x_2)$, and $f_4(x_4)$), we can identify favorable conditions for SM-cloud-precipitation feedback (SMCPF) within the height range of 1-3 km: (i) substantial boundary-layer moistening from wet soil (ii) the existence of a unstable lapse rate facilitated by a warm surface and a cool low-level atmosphere. This finding corroborates the physical mechanisms underlying SMCPF pathways (Wallace & Hobbs, 2006).

Further investigation of antecedent 7-hour TPW shows somehow the opposite pattern against AT. Such dependence of CVP on TPW can be explicated by its reflection of the

synoptic scale humidity of the early atmosphere. Intuitively, early TPW can be viewed as a proxy for the amount of water vapor that actually condenses and forms clouds and precipitation later. This is coordinated with the derived positive correlation between TPW and its component function, $f_5(x_5)$ in Figure S3c. In addition, TPW can be a precursor to mesoscale convective events. A sharp increase in TPW prior to the convective precipitation is indicative of the deep convection (Sherwood, 1999; Holloway & Neelin, 2010). This possibly explains why the magnitudes of $f_5(x_5)$ and its F-statistics increase with height so that CVP is more sensitive to TPW in the free atmosphere. 7-hour is observed to be the most informative time lag for the TPW-CVP relationship. This comes in excellent agreement with the conclusion of Holloway and Neelin (2010) that, with the involvement of mesoscale convective dynamics, a peak in TPW occurs typically 7-hour prior to the strong precipitation events at Nauru Island.

In summary, atmospheric controls on CVP can be altitude-dependent. The low-level AT, along with SM and LST, exhibits a governing effect on convective clouds/precipitation within the 1-3 km zone. TPW, by contrast, plays a critical role in shaping cloud and precipitation distribution in the free atmosphere.

Text S4. F-statistics of the Second-order Component Functions

We present in Figure S4 the F-statistic profiles for the second-order component function. As expected, most of them exhibit lower contributions to CVP than their first-order counterparts. This is particularly evident for the SM-related second-order component functions (see Figure S4a-d). The significance of the direct or main effect of the input variable is common in physical systems (Rabitz & Aliş, 1999; Ratto et al., 2007; Kucherenko et al., 2011; Gao et al., 2023). The LST-LAI component, $f_{23}(x_2, x_3)$, and the LAI-AT component, $f_{34}(x_3, x_4)$, exhibit relatively large mean F-statistics at 1-3 km. The joint contributions of LST-LAI and LAI-AT to the CVP could approximate the effects of plant transpiration and moistening of the boundary layer. However, we deliberately avoid offering detailed mechanistic interpretation as these F-statistics are accompanied by extremely high uncertainty. This uncertainty may stem from the pronounced west-to-east contrast in LAI over our study region. To be more specific, if the bootstrap method primarily samples convective precipitation events from the densely vegetated, humid eastern region, the influence of $f_{23}(x_2, x_3)$ and $f_{34}(x_3, x_4)$ on CVP could become more significant. On the contrary, in the western semi-arid region, where vegetation is sparse, these components have a diminished impact due to the reduced role of plant transpiration in regulating boundary layer moisture.

Text S5. Correlation Analysis and Multiple Linear Regression

Figure S5 displays vertical profiles of the Pearson correlation coefficient of DPR-measured cloud reflectivities and the different land-surface and atmospheric variables assembled in the 5×1 vector $\mathbf{x} = (\mathrm{SM}, \mathrm{LST}, \mathrm{LAI}, \mathrm{AT}, \mathrm{TPW})^\top$. Next, we use multiple linear regression and predict the DPR-measured cloud reflectivities, y, at each cloud height using the regression function, $y = f(\mathbf{x}, \boldsymbol{\beta}) = \beta_0 + \beta_1 x_1 + \ldots + \beta_5 x_5$. The least squares method offers a direct solution for the intercept $\widehat{\beta}_0$ and multiplicative coefficients $\widehat{\beta}_1, \ldots, \widehat{\beta}_5$. This multiple linear regression function ignores cooperative and interaction effects of the variables, but if these terms are deemed unimportant then the sign and magnitude of $\widehat{\beta}_1, \ldots, \widehat{\beta}_5$ should convey information about the contribution of SM, LST, LAI, AT, and TPW to the SMPFC. Figure S6 presents vertical profiles of the least squares coefficients $\widehat{\beta}_1, \ldots, \widehat{\beta}_5$, for SM time lag $\Delta t = 7$ (blue) and $\Delta t = 10$ (orange) hours. The coefficient profiles have a strong agreement with their counterparts of the correlation coefficient (Figure S5) but are not as consistent with the profiles obtained from the HDMR-F-statistic (Figure 2). We do not further discuss the similarities and differences between the profiles and methods. They all provide useful insights into the diurnal SMCPF. In general, the stronger the cooperative and/or interaction effects among the input variables, the greater the qualitative and quantitative differences are expected to be between HDMR and traditional regression methods. HDMR appears to better distinguish the characteristic time lags of the feedback, for instance, a 7-hour lag of the surface SM's feedback on cloud and precipitation at 1-3 km height. This is in agreement with Welty and Zeng (2018), who observed a statistically significant correlation between morning soil moisture anomalies and afternoon

precipitation in the Southern Great Plains. The LST profile obtained by HDMR further confirms this method's strength in capturing the physical dynamics of thermal updrafts over time, as discussed previously.

Text S6. Marshall-Palmer formula

The component function, $f_i(x_i)$ (dBZ), which quantifies the contribution of a variable (e.g., SM) to cloud reflectivity, can be further converted into estimates of rainfall rate through the Marshall-Palmer formula (Marshall & Palmer, 1948)

$$R_0 = \left[\frac{10^{(f_0/10)}}{200}\right]^{5/8} \tag{S6a}$$

$$R_1 = \left\{\frac{10^{[(f_0+f_i(x_i))/10]}}{200}\right\}^{5/8} \tag{S6b}$$

$$\Delta R = R_1 - R_0, \tag{S6c}$$

where R_0 signifies the mean rainfall rate (mm/hour) estimated from the mean cloud reflectivity, f_0 (dBZ), and R_1 is the same quantity but computed using the sum of mean cloud reflectivity and the SM component, $f_0 + f_i(x_i)$ (dBZ). By taking the difference between the two quantities (ΔR), we can readily determine the impact of SM on rainfall rates. According to Figure S7c, the 7-hour wet soil can account for up to a 2 mm/hour increment in rainfall rate at 2.0 km, denoting strong positive feedback.

References

Findell, K. L., & Eltahir, E. A. B. (2003a). Atmospheric controls on soil moisture–boundary layer interactions. Part I: Framework development. *Journal of Hydrometeorology*, *4*, 552-569. doi: 10.1175/1525-7541(2003)004⟨0552:ACOSML⟩2.0.CO;2

Findell, K. L., & Eltahir, E. A. B. (2003b). Atmospheric controls on soil moisture–boundary layer interactions. Part II: Feedbacks within the continental United States. *Journal of Hydrometeorology*, *4*(3), 570–583. doi: 10.1175/1525-7541(2003)004⟨0570:ACOSML⟩2.0.CO;2

Gao, Y., Sahin, A., & Vrugt, J. A. (2023). Probabilistic sensitivity analysis with dependent variables: Covariance-based decomposition of hydrologic models. *Water Resources Research*, *59*(4), e2022WR032834. Retrieved from `https://agupubs.onlinelibrary.wiley.com/doi/abs/10.1029/2022WR032834` doi: 10.1029/2022WR032834

Golub, G. H., & Van Loan, C. F. (1996). *Matrix computations (3rd ed.).* Baltimore: Johns Hopkins.

Holloway, C. E., & Neelin, J. D. (2010). Temporal relations of column water vapor and tropical precipitation. *Journal of the Atmospheric Sciences*, *67*(4), 1091–1105. doi: 10.1175/2009JAS3284.1

Iguchi, T., Seto, S., Meneghini, R., Yoshida, N., Awaka, J., Le, M., ... Kubota, T. (2010). GPM/DPR level-2 algorithm theoretical basis document. *NASA Goddard Space Flight Center*.

Kucherenko, S., Feil, B., Shah, N., & Mauntz, W. (2011). The identification of model effective dimensions using global sensitivity analysis. *Reliability Engineering & System Safety*, *96*(4), 440-449. doi: 10.1016/j.ress.2010.11.003

Li, G., & Rabitz, H. (2010). D-MORPH regression: application to modeling with unknown parameters more than observation data. *Journal of Mathematical Chemistry*, *48*, 1010–1035. doi: 10.1007/s10910-010-9722-2

Li, G., & Rabitz, H. (2012). General formulation of HDMR component functions with independent and correlated variables. *Journal of Mathematical Chemistry*, *50*(1), 99-130. doi: 10.1007/s10910-011-9898-0

Liao, L., & Meneghini, R. (2022). GPM DPR retrievals: Algorithm, evaluation, and validation. *Remote Sensing*, *14*(4), 843. doi: 10.3390/rs14040843

Marshall, J., & Palmer, W. M. (1948). The distribution of raindrops with size. *Journal of meteorology*, *5*, 166.

Penrose, R. (1955). A generalized inverse for matrices. *Proceedings of the Cambridge Philosophical Society*, *51*(3), 406-413. doi: 10.1017/S0305004100030401

Rabitz, H., & Aliş, Ö. F. (1999). General foundations of high-dimensional model representations. *Journal of Mathematical Chemistry*, *25*(2), 197-233. doi: 10.1023/A:1019188517934

Ratto, M., Pagano, A., & Young, P. (2007). State dependent parameter metamodelling and sensitivity analysis. *Computer Physics Communications*, *177*(11), 863-876. doi: 10.1016/j.cpc.2007.07.011

Reichle, R. H., Lucchesi, R. A., Ardizzone, J. V., Kim, G.-K., Smith, E. B., & Weiss, B. H.

(2015). *Soil moisture active passive (SMAP) mission level 4 surface and root zone soil moisture (L4_SM) product specification document* (Tech. Rep.). NASA Goddard Space Flight Center. Retrieved from `https://nsidc.org/sites/default/files/reichle789.pdf`

Sherwood, S. C. (1999). Convective precursors and predictability in the tropical western Pacific. *Monthly Weather Review*, *127*(12), 2977–2991. doi: 10.1175/1520-0493(1999)127⟨2977:CPAPIT⟩2.0.CO;2

Wallace, J. M., & Hobbs, P. V. (2006). *Atmospheric science: an introductory survey* (Vol. 92). Elsevier.

Welty, J., & Zeng, X. (2018). Does soil moisture affect warm season precipitation over the southern Great Plains? *Geophysical Research Letters*, *45*(15), 7866–7873. doi: 10.1029/2018GL078598

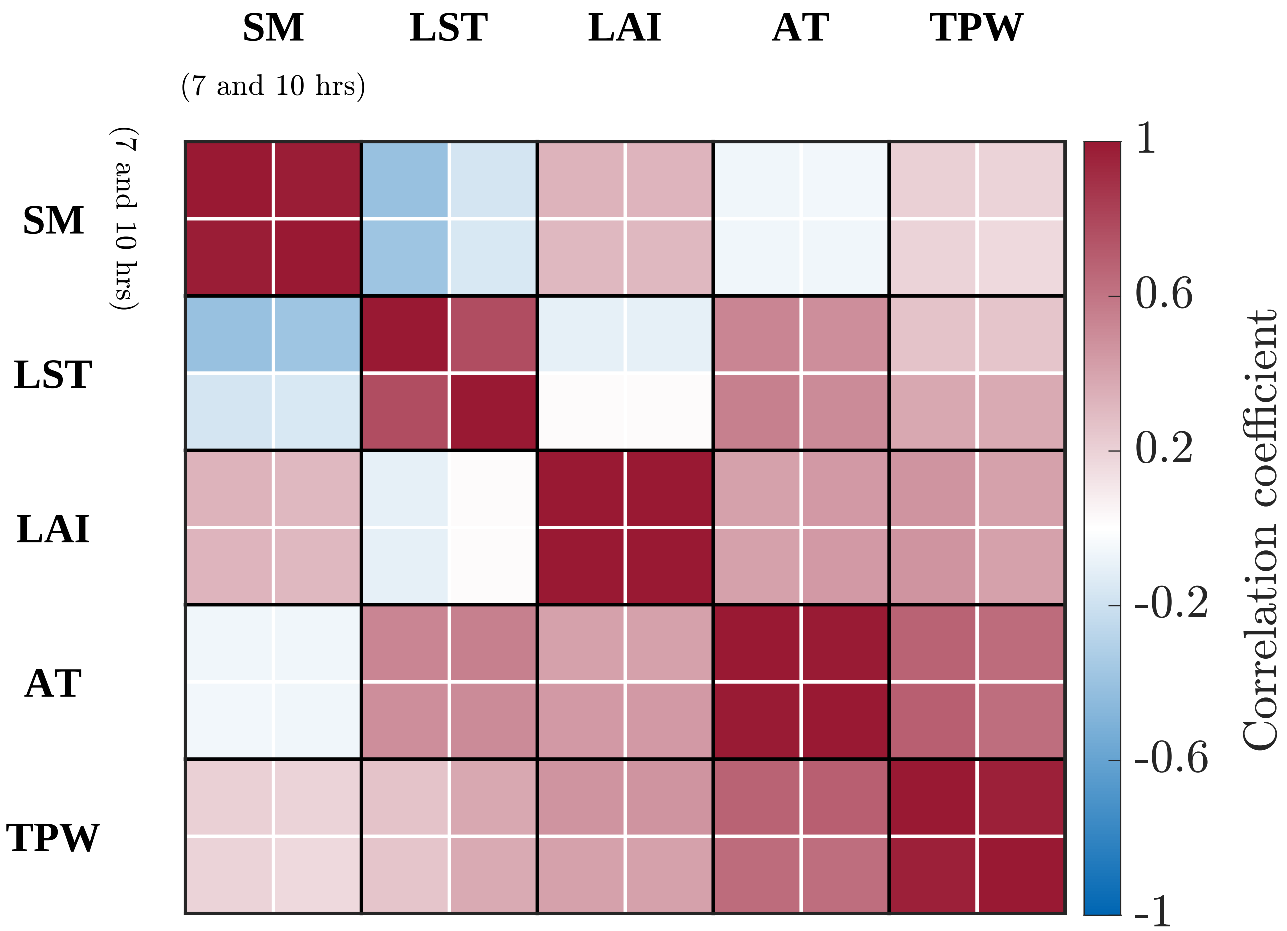

Figure S1. Correlogram of the land-surface and atmospheric variables used as inputs of HDMR. Solid black lines demarcate distinct variables, whereas solid white lines differentiate between time lags ($\Delta t = 7$ and 10 hours) relative to the DPR scanning time.

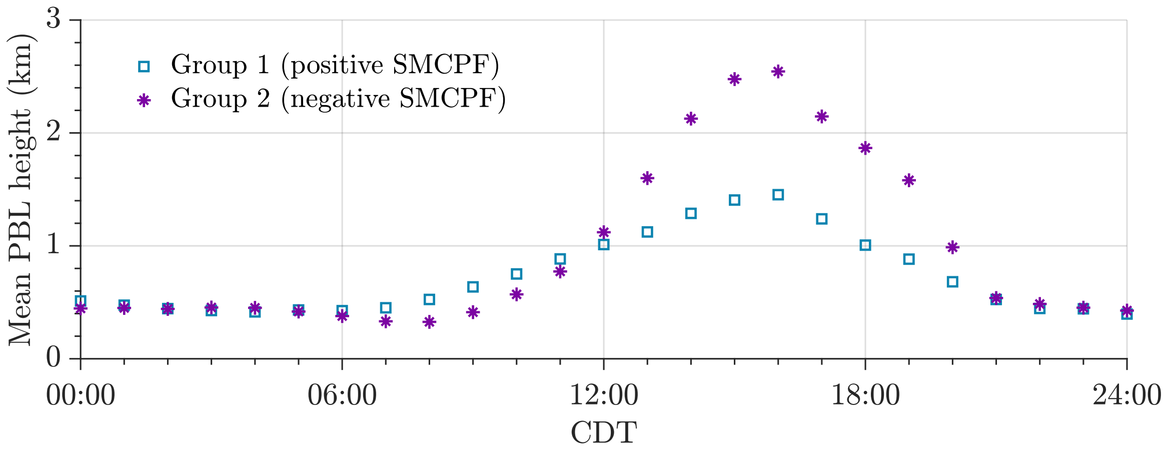

Figure S2. Diurnal development of the mean ERA5 reanalysis PBL height at Central Daylight Time (CDT) determined for two groups of samples. Groups 1 and 2 highlight positive SMCPF (blue squares) with $f_1(x_1) > 1.0$ dBZ and negative SMCPF (violet stars) with $f_2(x_2) > 1.0$ dBZ, respectively, both at a time lag of 7 hours and cloud height of 2.0 km.

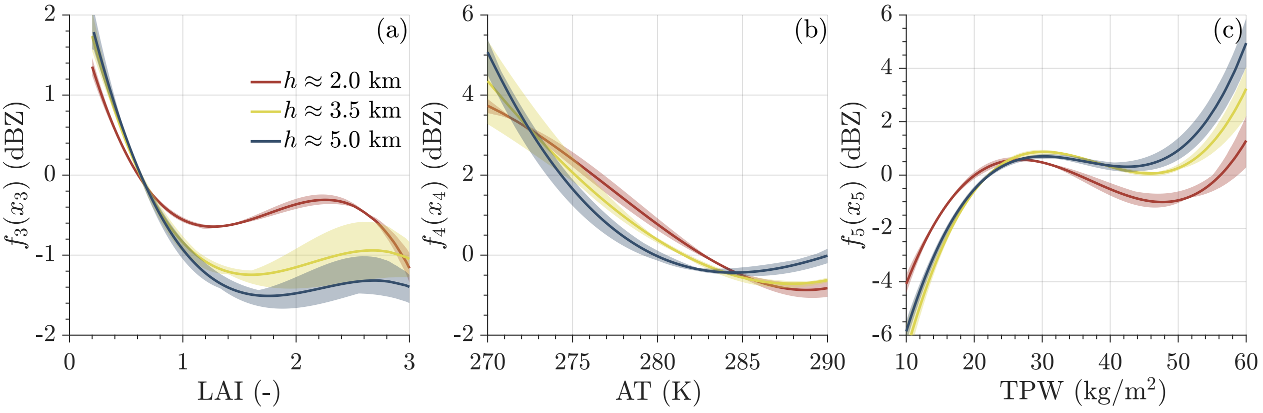

Figure S3. Antecedent 7-hour (a) LAI, (b) AT (K), and (c) TPW (kg/m^2) against their first-order component functions, $f_3(x_3)$ (dBZ), $f_4(x_4)$ (dBZ), and $f_5(x_5)$ (dBZ), respectively, evaluated at three separate heights, 2.0 km (red), 3.5 km (yellow), and 5.0 km (blue). The light-colored regions correspond to the 95% bootstrap confidence intervals.

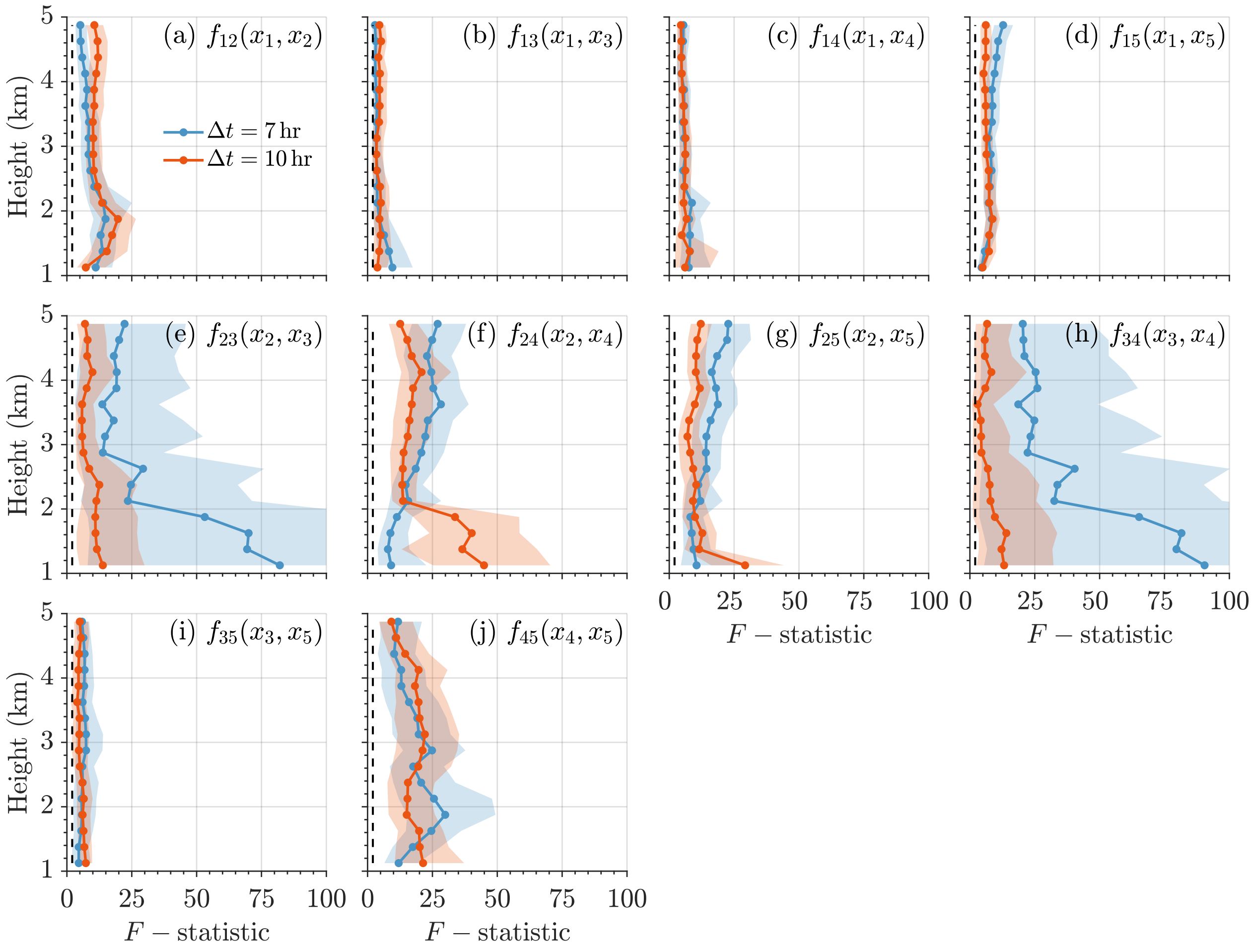

Figure S4. Same as Figure 2 in the main text but for the F-statistic profiles of the second-order component functions, $f_{ij}(x_i, x_j)$. Five land-surface and atmospheric variables are used in this study, $\mathbf{x} = (x_1, \ldots, x_5)^\top = (\mathrm{SM}, \mathrm{LST}, \mathrm{LAI}, \mathrm{AT}, \mathrm{TPW})^\top$, the pairs of which creates ten second-order component functions.

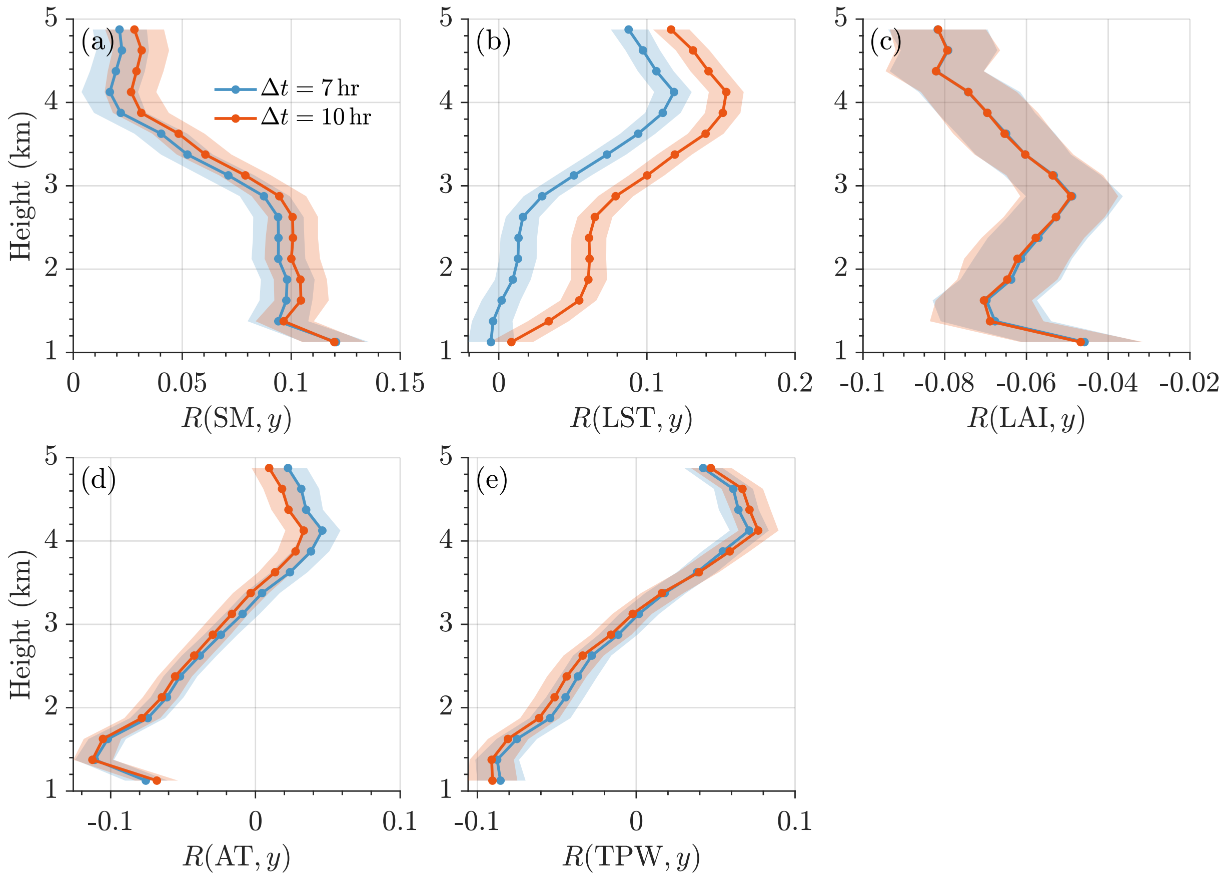

Figure S5. Same as Figure 2 in the main text but for the Pearson correlation coefficient between the cloud reflectivity profiles and each land-surface or atmospheric variable.

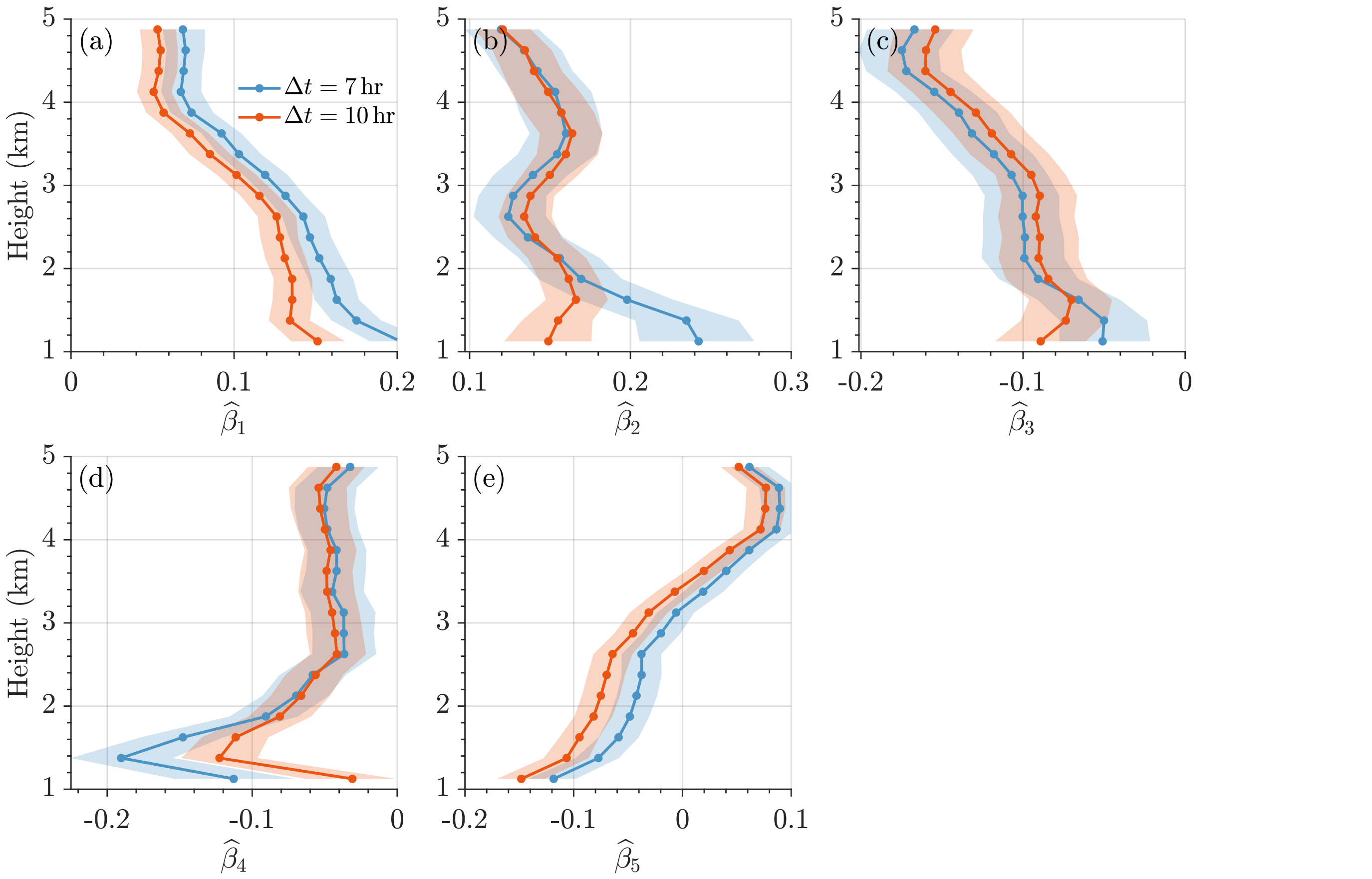

Figure S6. Same as Figure 2 in the main text but for the least squares coefficients $\widehat{\beta}_1, \ldots, \widehat{\beta}_5$. The coefficients are determined for a multiple linear regression function, $y = f(\mathbf{x}, \boldsymbol{\beta}) = \beta_0 + \beta_1 x_1 + \ldots + \beta_5 x_5$, at each separate cloud height and time lag.

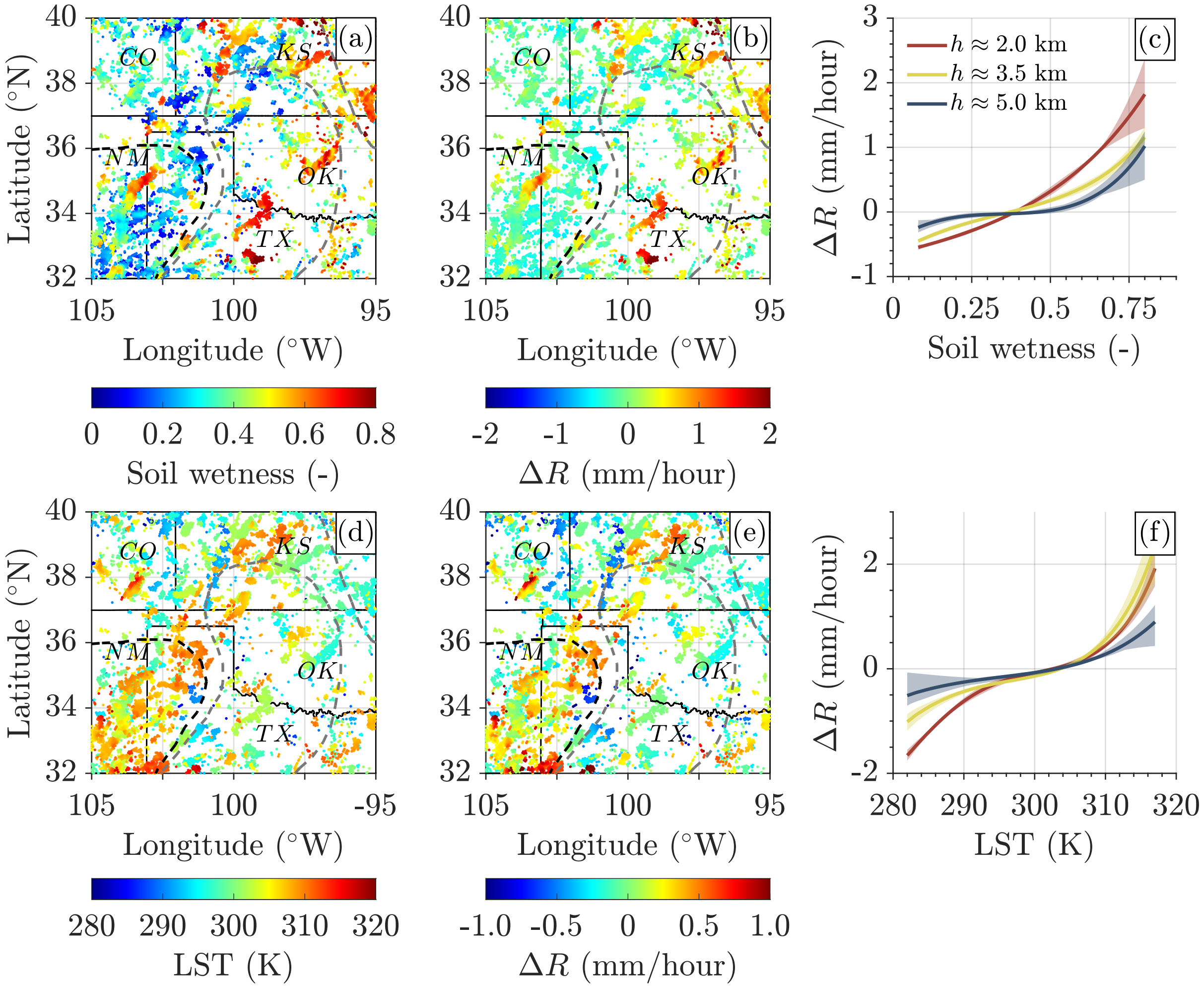

Figure S7. The central United States (95°W-105°W, 32°N-40°N) with (a) antecedent 7-hr SMAP/L4 soil wetness (-) of the top layer (0-5 cm) collocated at coordinates of the GPM/DPR/L2A samples and (b) change in rainfall rate, ΔR (mm/hour), at 2.0 km attributed to SM. Solid black lines delineate the state borders while dashed black and grey lines depict the negative feedback and transitional regions proposed by Findell and Eltahir (2003b). Panel (c) displays the scatter plots of the samples of antecedent 7-hour SM against the corresponding change in rainfall rate, ΔR (mm/hour), evaluated at three separate heights, 2.0 km (red), 3.5 km (yellow), and 5.0 km (blue). The bottom row of panels presents the same content as panels (a-c) but for (d) SMAP/L4 LST and (e,f) its associated impact on rainfall rate, ΔR (mm/hour).

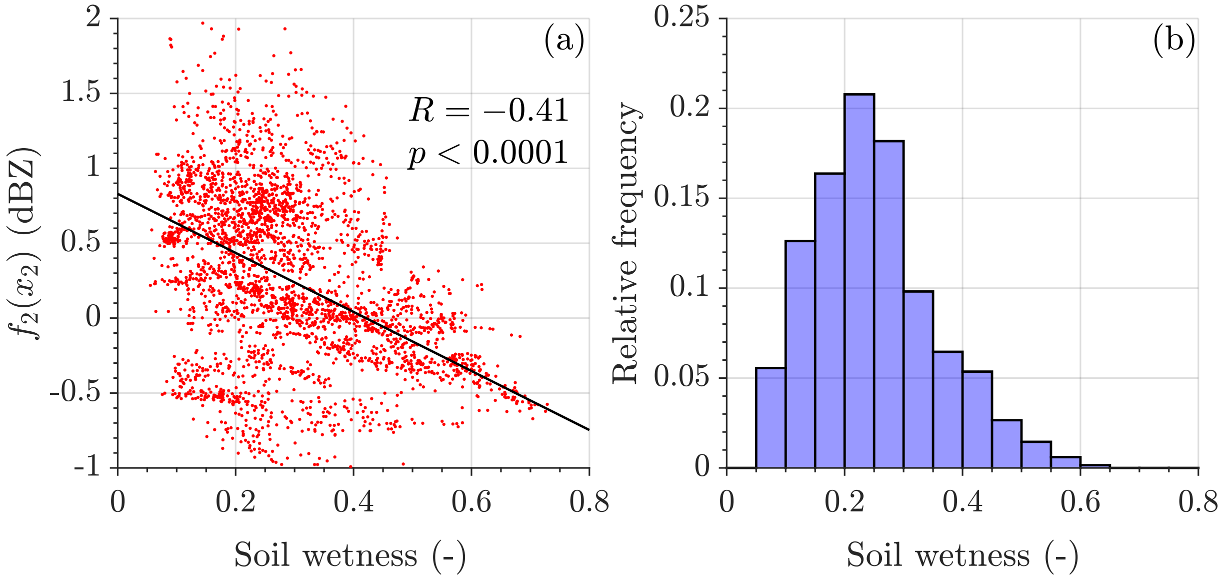

Figure S8. Evidence of negative SMCPF in the southwest (101°W-105°W, 32°N-36°N) of the central United States: (a) scatter plot of the SM samples from this area against the respective LST component function, $f_2(x_2)$; solid black line portrays the least squares fit of a simple regression function to the samples; (b) marginal distribution of SM subsampled from panel (a) with $f_2(x_2) > 0$ (dBZ). The negative correlation ($R = -0.41$) between SM and $f_2(x_2)$ and LST's pronounced contribution to the cloud over dry soils highlight the intrinsic SM-LST coupling. This aligns with the negative SMCPF.
